# In-Depth Understanding of *Camellia oleifera* Self-Incompatibility by Comparative Transcriptome, Proteome and Metabolome

**DOI:** 10.3390/ijms21051600

**Published:** 2020-02-26

**Authors:** Junqin Zhou, Mengqi Lu, Shushu Yu, Yiyao Liu, Jin Yang, Xiaofeng Tan

**Affiliations:** Key Laboratory of Cultivation and Protection for Non-Wood Forest Trees, Ministry of Education, Central South University of Forestry and Technology, Changsha 410001, China

**Keywords:** oil-tea, *Camellia oleifera*, mitogen-activated protein kinase, plant hormone, pollen tube death, programmed cell death, ubiquitination

## Abstract

Oil-tea tree (*Camellia oleifera*) is the most important edible oil tree species in China with late-acting self-incompatibility (LSI) properties. The mechanism of LSI is uncertain, which seriously hinders the research on its genetic characteristics, construction of genetic map, selection of cross breeding parents and cultivar arrangement. To gain insights into the LSI mechanism, we performed cytological, transcriptomic, proteomic and metabolomic studies on self- and cross-pollinated pistils. The studies identified 166,591 transcripts, 6851 proteins and 6455 metabolites. Transcriptomic analysis revealed 1197 differentially expressed transcripts between self- and cross-pollinated pistils and 47 programmed cell death (PCD)-control transcripts. Trend analysis by Pearson correlation categorized nine trend graphs linked to 226 differentially expressed proteins and 38 differentially expressed metabolites. Functional enrichment analysis revealed that the LSI was closely associated with PCD-related genes, mitogen-activated protein kinase (MAPK) signaling pathway, plant hormone signal transduction, ATP-binding cassette (ABC) transporters and ubiquitin-mediated proteolysis. These particular trends in transcripts, proteins and metabolites suggested the involvement of PCD in LSI. The results provide a solid genetic foundation for elucidating the regulatory network of PCD-mediated self-incompatibility in *C. oleifera*.

## 1. Introduction

Oil-tea tree (*Camellia oleifera*) is the most economically important forest species native to China [1]. The tree is cultivated for its seeds, which are the source of edible oil rich in unsaturated fatty acids and various active ingredients, such as squalene, tocopherol, vitamins, carotene, glycerol, triterpene alcohol and saponin, etc. [2,3]. Oil-tea camellia can be used for production of high value-added skin care products, cosmetics, Vaseline, soaps, shampoos and medical products such as oleic acid and its esters [4]. Although the flowering rate of *C. oleifera* is high, the fruit-setting rate is extremely low under natural conditions (less than 5%), mainly due to the genetic characteristics of self-incompatibility (SI) and improper pollinating cultivar arrangement. Understanding the mechanism of its SI system can potentially increase seed yield of *C. oleifera*.

SI is a precise genetic mechanism that prevents self-fertilization and promotes cross-pollination during the evolution of flowering plants [5,6,7]. It is estimated that about half of the flowering plants exhibit SI [8]. The research on the SI mechanism has become a frontier area in plant biology [9,10,11]. According to genetic characteristics and locations of pollen inhibition, plant SI is generally divided into three categories: sporophytic self-incompatibility (SSI), gametophytic self-incompatibility (GSI) and late-acting self-incompatibility (LSI). In the typical SSI plants *Brassica campestris* and *Brassica oleracea*, the SI reaction is dominated by the interaction between a stigma-specific expression receptor kinase (SRK) and a pollen-specific expression cysteine-rich protein (SCR) [12,13,14,15], followed by intracellular phosphorylation [16,17]. The GSI of Solanaceae, Scrophulariaceae and Rosaceae are regulated by S-RNase, which are determined by pistil-specific expression of S-RNase and pollen-specific expression of S-locus F-box protein (SLF/SFB) [18,19,20]. S-RNase can affect the degradation of pollen tube RNA and depolymerize the cytoskeleton, resulting in the occurrence of programmed cell death (PCD) in the pollen tube in *Pyrus bretschneideri* [21]. As S-RNase possesses cytotoxicity, it seems that S-RNase may trigger signaling pathways or defense responses following its entry into the pollen tube [21,22,23]. Contrary to the GSI and SSI systems where self-pollination pollen is stopped before reaching the ovary, a self-pollination pollen tube in LSI can grow smoothly in the style and reach the ovary, but in the end, it cannot be seeded or the seed-setting rate is significantly lower than that of the cross-pollinated plants [8]. A sizeable cohort of species present LSI property, but its molecular mechanism remains unclear [8,24].

LSI mechanisms can be studied through pollination biology, affinity index and fluorescence microscopic observation methods [25,26,27,28]. Molecular research also has been tried by screening for SI-related genes by RNA sequencing or genome-wide association study (GWAS). The knowledge of specifically or preferentially expressed genes of self-ovules (the ovule after self-pollination) is a valuable resource for genetic analyses of candidate genes involved in the LSI response; for example, Zhou and Zheng found that signal transduction-related genes and specifically expressed transcription factors might be involved in self-incompatible reactions in *Xanthoceras sorbifolium* [29]. Another study reported the transcriptome of styles after cross- and self-pollination and offered novel insights into the molecular mechanism of LSI in *Camellia sinensis* [30,31]. It was found that a single gene (CL25983Contig1) had high homology with S-RNase and was expressed at a significantly higher level 24 h after pollination in a self-pollinated plant than that in the cross-pollinated group [30]. Lanaud et al. used GWAS to screen loci associated with *Theobroma cacao* LSI and identified two loci located on chromosomes 1 and 4 that were closely related to the LSI reaction [32].

In *C. oleifera*, transmission and scanning electron microscopy had shown that the pollen tube reached the base of the style in the oil-tea tree self-pollination process, then the tube walls were thickened and the disintegration of the organelle in the wall was unrecognizable [33], which are PCD characteristics [34]. The process of inhibiting pollen tube growth in *C. oleifera* was similar to that of *Pyrus pyrifolia* and *Malus domestica* in Rosaceae with the GSI system, except that the location where the pollen tubes were arrested was different. However, the SI mechanism of *C. oleifera* may be much more complicated due to the complex ploidy (2n = 6x). The lack of complete genome sequencing of *C. Oleifera* has greatly restricted the basic research of oil-tea trees at the molecular level.

RNA-Seq technology generates short-length sequencing reads. These short sequences do not assemble full-length transcripts but provide high sequencing depth and sequence correctness [35]. The recently developed Pacific Biosciences long-read transcriptome sequencing technology (Iso-Seq) can capture a complete catalogue of transcripts and their variants [36,37] but with higher error rate, which can be corrected by short and high-precision RNA-seq reading [38,39,40]. The combination of the second- and third-generation transcriptomics can provide both high-quality transcripts and the expression levels of transcripts in specific samples, which is of great significance for studying key genes and metabolic pathways involved in LSI of *C. Oleifera*. Taking advantage of transcriptome, proteome and metabolome analysis, this study was designed to use samples of self- and cross-pollinated pistils to screen for key genes, proteins and metabolites during the process of LSI in *C. oleifera*. This data should provide a solid foundation for elucidating the LSI molecular mechanism of *C. oleifera*.

## 2. Results

### 2.1. Cytological Observation of SI Type in C. Oleifera

**Figure 1:**
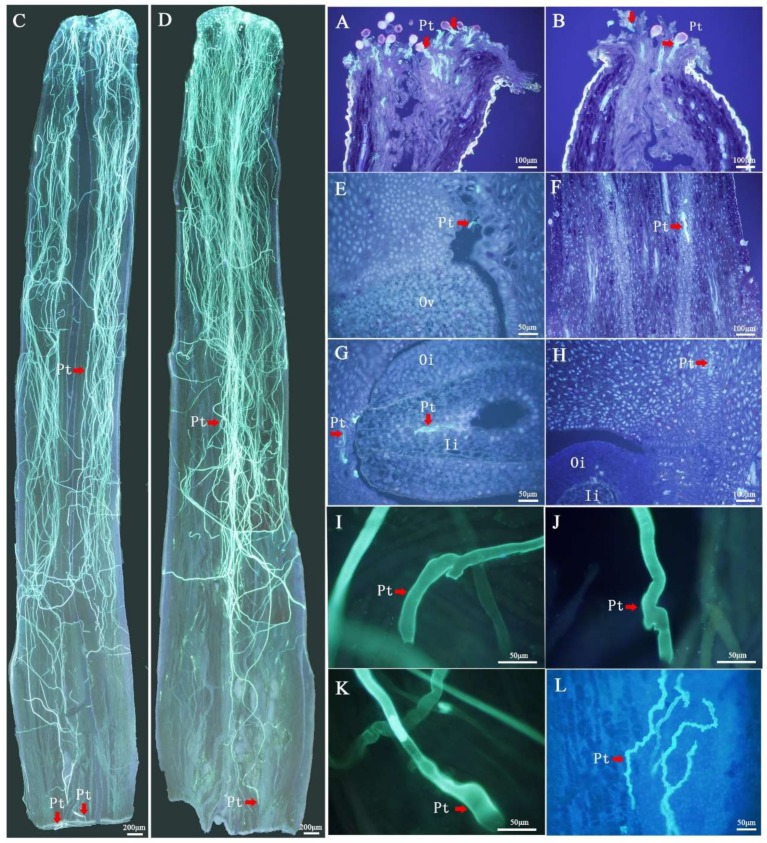
Pollen tube growth from 2 h to 72 h in the pistil after self- and cross-pollination in *Camellia oleifera*. (**A**) Pollen grain germination on stigma at 2 h after cross-pollination (CP) (100×). (**B**) Pollen grain germination on stigma at 2 h after self-pollination (SP) (100×). (**C**) Pollen tube growth to the base of the style at 48 h after CP (40×). (**D**) Pollen tube growth to the base of the style at 48 h after SP (40×). (**E**) Pollen tube growth to the upper part of ovary at 48 h after CP (200×). (**F**) Pollen tube growth to ovary at 48 h after SP (100×). (**G**) Pollen tube growth to ovule at 72 h after CP (200×). (**H**) Pollen tube growth to ovary at 72 h after SP (100×). (**I**–**K**) Abnormally shaped pollen tubes at the base of style after SP. (**L**) Abnormally shaped pollen tube at the upper part of ovary after SP. Pt = pollen tube, Es = embryo sac, Oi = outer integument and Ii = inner integument.

During the first 48 h after pollination, the pollen tube growth rate was basically the same between the self- and cross-pollinated plants (Figure 1A–F). However, after 48 h, the growth rate of the self-pollinated pollen tubes was significantly lower than that of the cross-pollinated. The self-pollinated pollen tubes stopped growing between 48 and 72 h after pollination, and the LSI reactions appeared, such as tissue distortion and folding (Figure 1I–L). It had been found that LSI reactions in *C. Oleifera* were caused by abnormal thickening of the pollen tube wall with unidentifiable organelle [33], which are the characteristics of PCD [34]. In the meantime, the pollen tubes entered the ovule by micropyle at 72 h after cross-pollination (Figure 1G). These observations support the classification of *C. Oleifera* SI as LSI.

### 2.2. Illumina- and SMRT-Based RNA Sequencing and Error Correction

**Figure 2:**
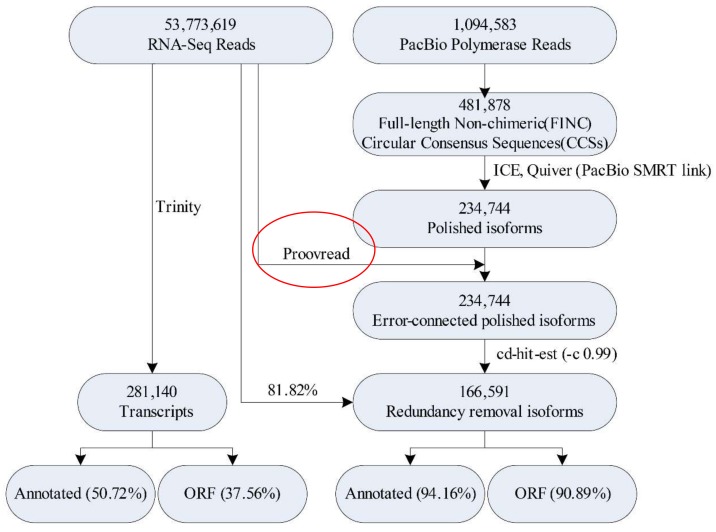
The analysis process of Illumina-seq and SMRT-seq in *Camellia oleifera*. ORF—open reading frame.

Illumina- and single molecule real-time (SMRT)-seq platforms were used to perform in-depth analysis of gene expression during oil-tea camellia LSI (Figure 2). To identify as much longer transcripts as possible, equal amounts of total RNA from each sample were pooled together and reverse-transcribed for SMRT-seq. Total RNA with OD_260/280_ ≥ 1.81, RIN ≥ 9.4 and 28S/18S ≥ 1.5 were used to build libraries (Table S2). To minimize bias that favors sequencing of shorter transcripts, two size-fractionated cDNAs (>2 kb and <2 kb) were constructed and subsequently sequenced in two SMRT cells (Table S3). A total of 481,878 full-length non-chimeric transcripts were identified, which accounted for 88.3% of reads of insert (ROIs) (Figure S1A). Finally, correction of the polished isoform sequences from the second-generation sequencing data was done with proofreading error correction software [39]. Using 166,591 redundant transcripts as the reference sequence for Illumina-seq, the total mapping rate was 81.82% (Figure 2). The PCA diagram of the transcriptome showed that the samples were tightly clustered together, indicating the reliability of the experiment and the reasonable selection of the samples (Figure S1F).

In order to better understand the advantages of the combination of Illumina- and SMRT-based RNA sequencing, we compared the Illumina de novo assembled transcripts (281,140) to the PacBio Iso-Seq consensus transcripts (166,591) (Figure S2). The mean length (1896 bp) and N50 (2198 bp) were longer among the Iso-Seq consensus transcripts than those among the Illumina RNA-seq data (Figure S2A). Moreover, Iso-Seq technology yielded more contiguous transcripts and contained a higher proportion of intact annotation rate (94.16%) and open reading frames (ORFs) (90.89%) than those only using the Illumina method (Figure 2 and Figure S2B–F). 

### 2.3. Functional Annotation and Categorization of Transcripts

To predict the functions of the 166,591 transcripts, we used software Diamond to perform functional annotation using NR, GO, KEGG, KOG and Swiss-Prot. A total of 132,738 transcripts were successfully matched to known proteins in at least one of the five databases, and 33,612 transcripts received high scores with known proteins in all five databases (Figure S1B).

To classify the functions of the *C. oleifera* transcripts, assignment of GO terms was performed using software Diamond from the NR database. In total, 111,856 transcripts were matched to GO terms, which were classified into three major functional categories, including biological process, cellular component and molecular function (Figure S1C). A total of 77,810 transcripts were predicted using the KEGG database, which were divided into five branches (cellular processes, environmental information processing, genetic information processing, metabolism and organismal systems) (Figure S1D). According to the KOG database, 68,629 transcripts participated into 25 functional groups (Figure S1E).

### 2.4. Transcript Expression Profiles and Enrichment in Self- and Cross-Pollinated Plants

**Figure 3:**
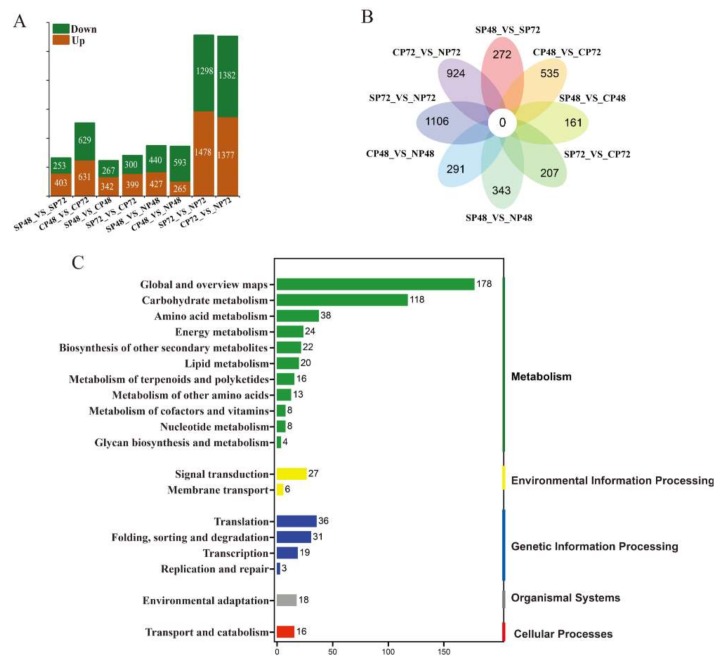
Analysis of differentially expressed transcripts (DETs) in self-, cross- and non-pollinated pistils of *Camellia oleifera*. (**A**) Numbers of DETs in the 8 pairs of self-, cross- and non-pollinated pistils. (**B**) Venn-flower diagram of DETs in the 8 pairs of self-, cross- and non-pollinated pistils. (**C**) KEGG pathway annotation of DETs in self- and cross-pollinated pistils. SP48/72, self-pollinated pistils at 48 h/72 h; CP48/72, cross-pollinated pistils at 48 h/72 h and NP48/72, non-pollinated pistils at 48 h/72 h.

There were 656, 1260, 609, 699, 867, 858, 2776 and 2759 differentially expressed transcripts (DETs) in the eight pairs of sample comparisons SP48_vs_SP72, CP48_vs_CP72, SP48_vs_CP48, SP72_vs_CP72, SP48_vs_NP48, CP48_vs_NP48, SP72_vs_NP72 and CP72_vs_NP72, respectively (Figure 3A). There were no common DETs in all of the eight comparison groups (Figure 3B). We identified 1197 DETs between self- and cross-pollinated pistils (Table S4), including 342 significantly up-regulated transcripts in SP48_vs_CP48 and 399 in SP72_vs_CP72 and 267 significantly downregulated transcripts in SP48_vs_CP48 and 300 in SP72_vs_CP72 (Figure 3A). According to the Venn diagram of the DETs, there were 368 specific DETs in self- and cross-pollinated pistils (Figure 3B). KEGG annotation showed that 1197 DETs were enriched in 19 classes of KEGG pathways and classified in five groups (Figure 3C). The significantly enriched pathways included pentose and glucuronate interconversions and ascorbate and aldarate metabolism in the carbohydrate metabolism class, biosynthesis of amino acids in the amino acids metabolism class, carbon fixation in photosynthetic organisms in the energy metabolism class, ATP-binding cassette (ABC) transporter in the membrane transport class, mitogen-activated protein kinase (MAPK) signaling pathway plant and plant hormone signal transduction in the signal transduction class, aminoacyl-tRNA biosynthesis in the translation class and ubiquitin-mediated proteolysis in the folding, sorting and degradation class.

**Figure 4:**
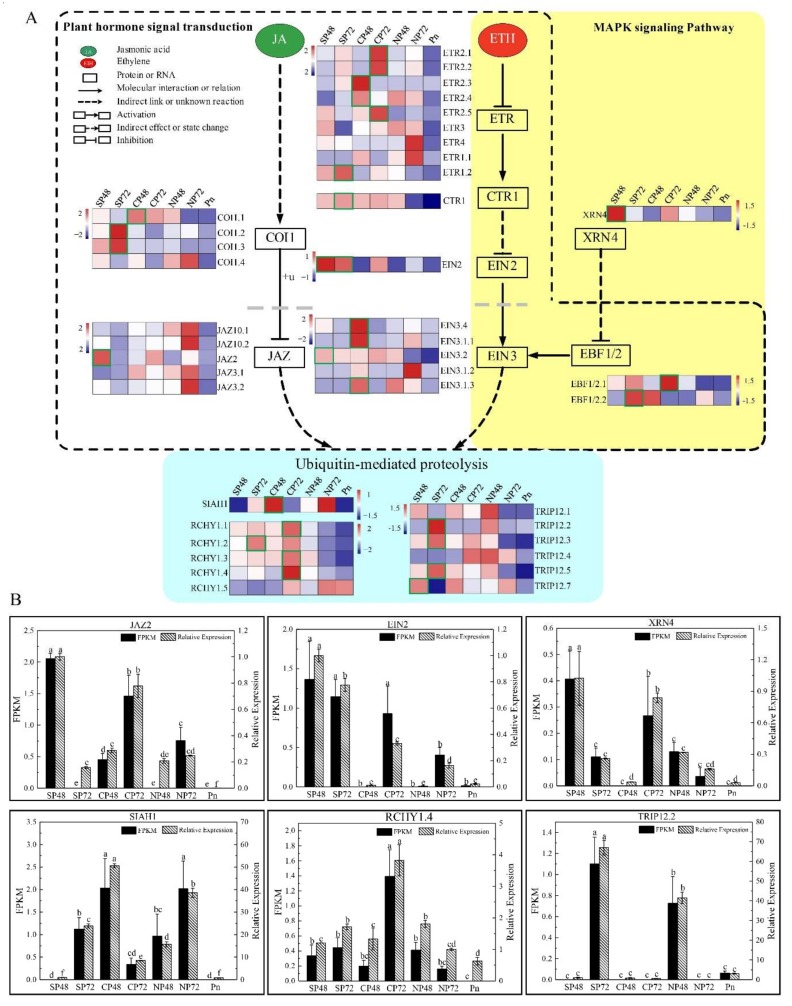
Metabolic pathways involved in self-incompatibility of *Camellia oleifera*. (**A**) Expression levels of each transcript in self-, cross- and non-pollinated pistils and mature pollen were shown by heatmap using log10(FPKM). FPKM = fragments per kilobase per million mapped fragments. The dotted line represents plant hormone signal transduction, the yellow background represents the plant MAPK signaling pathway and the blue background represents the ubiquitin-mediated proteolysis. The rectangles marked with green frames represent transcripts with significant differences in SP48_VS_CP48 or SP72_VS_CP72 by Duncan’s test. SP48/72, self-pollinated pistils at 48 h/72 h; CP48/72, cross-pollinated pistils at 48 h/72 h; NP48/72, non-pollinated pistils at 48 h/72 h and Pn, mature pollen. COI1: coronatine insensitive 1 (*COI1.1*: cb10019_c10797/f1p1/2331, *COI1.2*: cb10019_c12051/f3p2/2439, *COI1.3*: cb10019_c213631/f7p3/2189 and *COI1.4*: cb10019_c24162/f1p1/2313); JAZ: jasmonate ZIM-domain protein (*JAZ10.1*: cb10082_c1/f6p0/1155, *JAZ10.2*: cb10082_c9/f1p0/2636, *JAZ2*: cb15491_c52/f1p1/1212, *JAZ3.1*: cb15491_c53/f1p0/995 and *JAZ3.2*: cb15491_c77/f2p0/1218); ETR: ethylene receptor (*ETR2.1*: cb17705_c11/f2p0/917, *ETR2.2*: cb17705_c23/f1p0/3523, *ETR2.3*: cb17705_c46/f1p0/2261, *ETR2.4*: cb17705_c49/f1p1/3042, *ETR2.5*: cb17705_c50/f1p0/3281, *ETR3*: cb2257_c41/f1p0/1264, *ETR4*: cb2257_c48/f1p0/5354, *ETR1.1*: cb2257_c52/f6p0/3288 and *ETR1.2*: cb5372_c7/f2p0/2692); CTR1: serine/threonine-protein kinase 1 (*CTR1*: cb13260_c20799/f1p0/1502); EIN2:ethylene-insensitive protein 2 (*EIN2*: cb18338_c4/f1p0/1901); EIN3: EIN3-like protein (*EIN3.4*: cb16581_c3/f1p0/1238, *EIN3.1.1*: cb3510_c25/f3p0/1874, *EIN3.2*: cb3510_c34/f1p0/2313, *EIN3.1.2*: cb3510_c58/f2p0/1728 and *EIN3.1.3*: cb3510_c60/f1p1/1582); XRN4: 5′-3′ exoribonuclease 4 (*XRN4*: cb10019_c84410/f1p0/2840); EBF1/2: EIN3-binding F-box protein (*EBF1/2.1*: cb2074_c31/f2p0/2581 and *EBF1/2.2*: cb2074_c97/f4p3/2739); SIAH1: E3 ubiquitin-protein ligase SIAH1 (*SIAH1*: cb3579_c35/f1p0/1059); RCHY1: RING finger and CHY zinc finger domain-containing protein 1 (*RCHY1.1*: cb10019_c30957/f9p2/3704, *RCHY1.2*: cb10019_c40423/f1p0/2236, *RCHY1.3*: cb10019_c49228/f3p1/2088, *RCHY1.4*: cb10019_c49752/f1p0/2425 and *RCHY1.5*: cb15316_c3/f2p0/1121) and TRIP12: E3 ubiquitin-protein ligase UPL4 (*TRIP12.1*: cb10019_c108175/f3p0/2352, *TRIP12.2*: cb10019_c2620/f4p1/3672, *TRIP12.3*: cb10019_c2623/f1p0/2608, *TRIP12.4*: cb16096_c25/f4p0/1881, *TRIP12.5*: cb16096_c9/f1p0/1267 and *TRIP12.7*: cb19931_c8/f2p0/2614). The numbers behind the transcript name represent the transcript ID in the transcriptome. (**B**) Expression levels of *JAZ2*, *EIN2*, *XRN4*, *SIAH1*, *RCHY1.4* and *TRIP12.2* based on FPKM and qRT-PCR data. SP48 sample was the reference to calculate the relative expression data. Error bars indicate SD. Different letters represent significant difference at mRNA levels (*p* < 0.05).

Hormone signal transduction and MAPKs are associated with responses to SI [22,29,30]. A previous study provided evidence that MAPKs could induce PCD [41,42,43]. In our results, DETs in the self- and cross-pollinated pistils were significantly enriched in plant hormone signal transduction, MAPK signal transduction pathways and the ubiquitin-mediated proteolysis pathway (Figure 4A). As shown in Figure 4A, there were 27 transcripts (*COI1s*, *JAZs*, *ETRs*, *CTR1*, *EIN2*, *EIN3s* and *EBF1/2s*) encoding JA and ETH-signaling-related components and 18 transcripts (*ETRs*, *CTR1*, *EIN2*, *EIN3s* and *EBF1/2s*) encoding MAPK-signaling-related components. The expression of *XRN4* was observed predominantly in SP48, and the encoded protein was involved in the degradation of de-capped mRNAs, nonsense mediated decay, microRNA decay and essential for proper development [44]. *RCHY1.2*, *TRIP12.2, TRIP12.3* and *TRIP12.5* had relatively high expression levels in SP72; the expression levels of *STAH1* was higher in CP48 and *RCHY1.1*, *RCHY1.3* and *RCHY1.4* were more highly expressed in CP72 than other combinations, which were three types of E3 ubiquitin ligase related to ubiquitin-mediated proteolysis [45]. The qRT-PCR analysis of six transcripts (*JAZ2*, *EIN2*, *XRN4*, *SIAH1*, *RCHY1.4* and *TRIP12.2*) revealed consistent expression patterns with those generated by RNA-seq data (Figure 4B). Moreover, calmodulin *CaM* (cb21070_c0/f2p0/995)-encoding proteins involved in MAPK signal transduction and the expression level of *CaM* in CP72 was 23.7 times than that in SP72. In addition, there were many differentially expressed transcription factors in self- and cross-pollinated pistils, for example, *WRKY33* (cb8503_c4/f1p0/2186) and *B-ARR* (cb1922_c15/f1p0/2614) had significantly higher expression in CP72 than in SP72. The two transcripts code for proteins related to MAPK signaling pathway and plant hormone signal transduction, respectively. These gene products could be related to LSI in *C. oleifera*. 

### 2.5. Identification of PCD Candidate Transcriptsin Self- and Cross-Pollination

**Figure 5:**
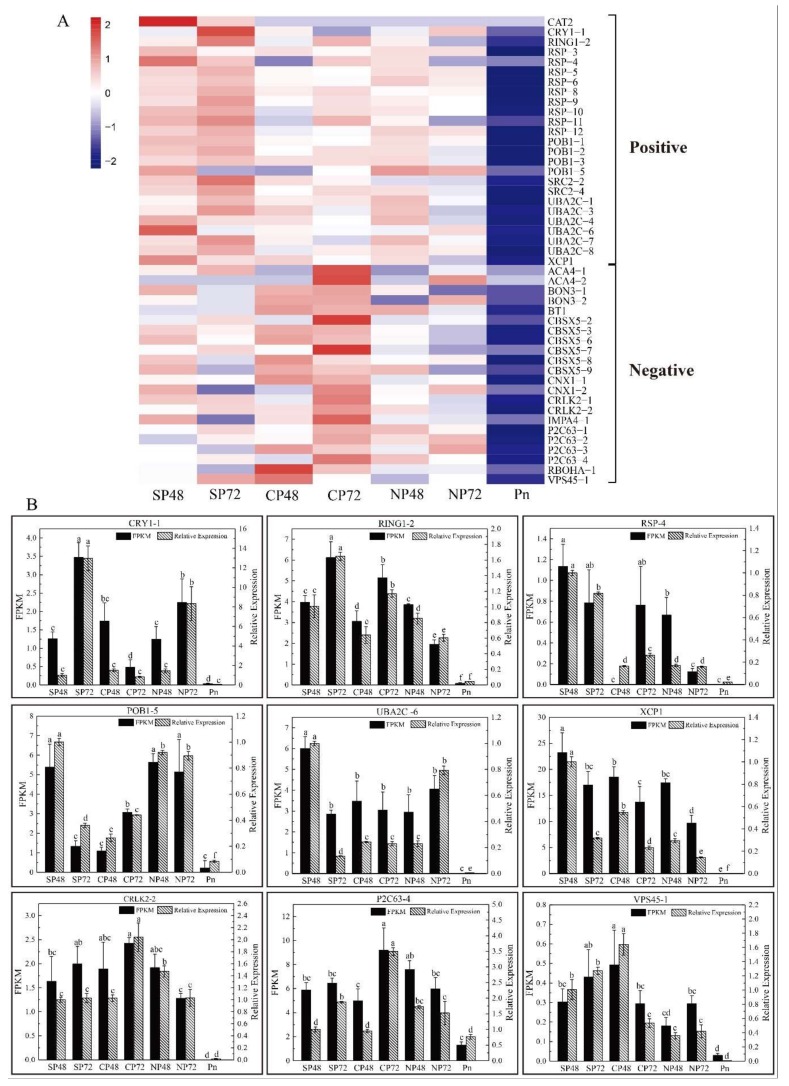
Expression patterns of programmed cell death (PCD)-related genes in self-, cross- and non-pollinated pistils and mature pollen of *Camellia oleifera*. (**A**) The heatmap of PCD-related genes in self-, cross- and non-pollinated pistils and mature pollen. Positive refers to positive-regulated transcripts. Negative refers to negative-regulated transcripts. SP48/72, self-pollinated pistils at 48 h/72 h; CP48/72, cross-pollinated pistils at 48 h/72 h; NP48/72, non-pollinated pistils at 48 h/72 h and Pn, mature pollen. *CAT2*, catalase isozyme 2; *CRY1*, cryptochrome-1; *RING1*, E3 ubiquitin-protein ligase RING1; *RSP*, CO(2)-response secreted protease; *SRC2*, SRC2 homolog; *UBA2C*, UBP1-associated protein 2C; *XCP1*, xylem cysteine proteinase1; *ACA4*, alpha carbonic anhydrase 4; *BON3*, BONZAI 3; *BT1*, adenine nucleotide transporter BT1; *CBSX5*, CBS domain-containing protein CBSX5; *CNX1*, molybdopterin adenylyltransferase; *CRLK2*, leucine-rich repeat receptor-like serine/threonine-protein kinase CRLK2; *IMPA4*, importin subunit alpha-4; *P2C63*, phosphatase 2C 63; *RBOHA*, respiratory burst oxidase homolog protein A and *VPS45*, vacuolar protein sorting-associated protein 45. (**B**) Expression level of *CRY1-1*, *RING1-2*, *RSP-4*, *POB1*, *UBA2C-6*, *XCP1*, *CRLK2-2*, *P2C63-4* and *VPS45-1* based on FPKM and qRT-PCR data. FPKM = fragments per kilobase per million mapped fragments. SP48 sample was the reference to calculate the relative expression data. Error bars indicate SD. Different letters represent significant difference at mRNA levels (*p* < 0.05).

Cytological observation showed that *C. oleifera* pollen tube growth inhibition (Figure 1) after self-pollination is PCD-related [33]. Twenty putative PCD-related genes (10 positive and 10 negative) from literatures [46,47,48,49,50,51] were identified. In Table S5, we can find that 73 PCD-related transcripts were expressed in mature pollen at lower levels than those in self-, cross- and non-pollinated pistils. Forty-seven transcripts showed significant differences in SP and CP by Duncan’s test (Figure 5A). Most of the positive genes exhibited relatively high levels of expression in self-pollinated pistils, such as catalase isozyme 2 (*CAT2*), cryptochrome-1 (*CRY1*), E3 ubiquitin-protein ligase (*RING1*), CO(2)-response secreted protease (*RSP*) and UBP1-associated protein 2C (*UBA2C*). Relatively, the expression levels of negative genes were lower after self-pollination and higher in cross- or non-pollinated pistils, such as alpha carbonic anhydrase 4 (*ACA4*), BONZAI 3 (*BON3*), CBS domain-containing protein (*CBSX5*) and respiratory burst oxidase homolog protein A (*RBOHA*). The qRT-PCR analysis of nine transcripts revealed consistent expression patterns with those generated by RNA-seq data (Figure 5B). These expression patterns suggested that the products of these PCD-related genes could have important roles in the inhibition of pollen tube growth of *C. oleifera.*

### 2.6. Proteomic Changes of C. oleifera in Response to SI

**Figure 6:**
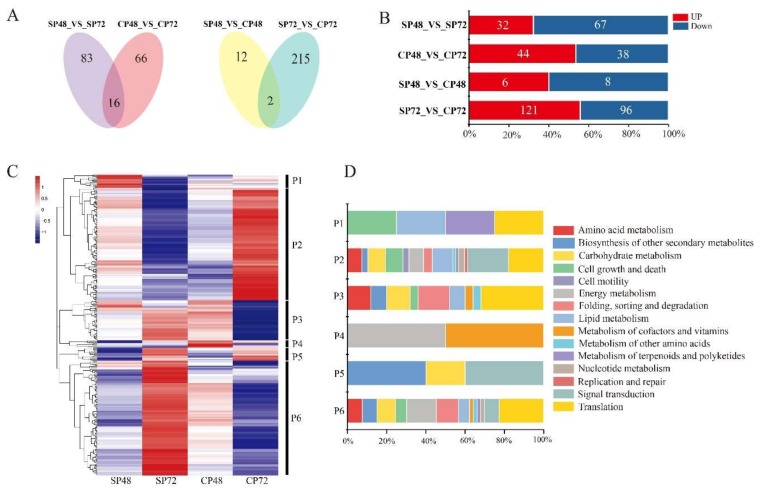
Differentially expressed proteins (DEPs) analysis of *C. oleifera* after self- and cross-pollination. (**A**) Venn diagrams of DEPs in self- and cross-pollinated pistils at 48 and 72 hours. SP48/72, self-pollinated pistils at 48 h/72 h and CP48/72, cross-pollinated pistils at 48 h/72 h. (**B**) Numbers of DEPs in self- and cross-pollinated pistils. (**C**) Hierarchical clustering analysis of 295 DEPs after self- and cross-pollination of *C. oleifera*. DEPs clustered into six groups (P1–P6). (**D**) Stacked bar chart of classification of KEGG pathway in six groups. The X-axis represents the proportion of each class in the corresponding cluster.

The PCA diagram of proteome showed that the samples were tightly clustered together, indicating that the high repeatability of the proteome experiment (Figure S3). A total of 6851 proteins were identified in the pistils after self- and cross-pollination of *C. oleifera*, which accounted for 4.52% of total predicted proteins by transcriptome analysis. There were 294 differentially expressed proteins (DEPs) in the four combinations, including SP48_vs_SP72, CP48_vs_CP72, SP48_vs_CP48 and SP72_vs_CP72 (Table S6). The results of the Venn chart showed 229 DEPs in SP48_vs_CP48 and SP72_vs_CP72 (Figure 6A), including 6 and 121 up-regulated proteins in SP48_vs_CP48 and SP72_vs_CP72, respectively, and 8 and 96 downregulated proteins in SP48_vs_CP48 and SP72_vs_CP72, respectively (Figure 6B).

The 294 DEPs were functionally categorized into 15 classes (Figure 6D). Other than proteins of unknown function (54.1%), the most abundant classes were translation (11.1%), followed by signal transduction (6.8%); carbohydrate metabolism (5.1%); energy metabolism (4.8%); lipid metabolism (4.5%); folding, sorting and degradation (4.5%) and cell growth and death (3.8%).

Furthermore, hierarchical cluster analysis of the 294 DEPs identified six distinct clusters based on the synthesis patterns of the proteins in self- and cross-pollinated pistils (P1–P6 in Figure 6C). Cluster 1 (P1) contained 13 proteins, 10 of which were unknown proteins, and the other three were xanthoxin dehydrogenase (ABA2, cb19839_c2/f1p0/1055), transformer-2 protein (TRA2, cb9377_c1/f6p0/1074 ) and tubulin alpha (TUBA, cb13260_c12875/f1p0/1873), which were highly synthesized in SP48 than in other combinations. Cluster 2 (P2) contained 110 proteins in 14 classifications, which were highly produced at CP72 and less produced at SP72. This cluster included transcription factor TGA (TGA, cb957_c24/f1p0/1843) and phosphatidylinositol phospholipase C (PLCD, cb10019_c291819/f1p0/2084), which are involved in plant hormone signal transduction and calcium signal transduction. Moreover, profilin (PFN, cb9129_c2/f7p0/760) and mitogen-activated protein kinase 1/3 (MAPK1/3, cb20236_c1/f1p0/1645) only existed in Cluster 2, which participated in regulation of actin cytoskeleton. The proteins in Cluster 3 (P3) were mainly synthesized at SP72 and CP48, and the proteins in Cluster 4 (P4) were mainly synthesized at CP48. Cluster 5 (P5) contained 13 proteins, which were mainly synthesized at SP72 and CP72. Cluster 6 (P6) contained 112 proteins in 13 classifications, which were highly synthesized at SP72 and less synthesized at CP72. Interestingly, Cluster P6 included splicing factor 3B subunit 4 (SF3B4, cb7943_c15/f7p0/1408); translation initiation factor 4A (EIF4A, cb14714_c13/f6p0/1668); ubiquitin-activating enzyme E1 (UBE1, cb10019_c268110/f1p1/3062) and WD repeat-containing protein 61 (WDR61, cb950_c10/f2p1/3475), which are related to MAPK signal transduction, ubiquitin-mediated proteolysis and RNA degradation. These proteins or enzymes might be involved in response to LSI of *C. oleifera*.

### 2.7. Overall Metabolite in Different Groups of C. Oleifera

**Figure 7:**
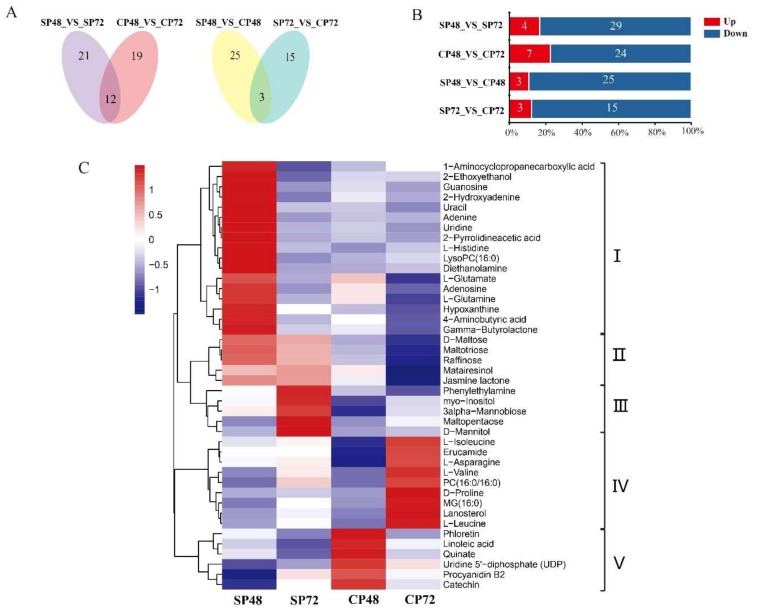
Differentially expressed metabolites (DEMs) analysis of *C. oleifera* after self- and cross-pollination. (**A**) Venn diagrams of DEMs in self- and cross-pollinated pistils at 48 and 72 h. (**B**) Numbers of DEMs in self- and cross-pollinated pistils. (**C**) Hierarchical clustering analysis of DEMs after self- and cross-pollination of *C. oleifera*. SP48/72, self-pollinated pistils at 48 h/72 h and CP48/72, cross-pollinated pistils at 48 h/72 h.

The PCA diagram of metabolome showed that each group had six replicates; one of the SP48 replicates and one of the SP72 replicates were separated from the rest, which were tightly clustered together in (Figure S4). We deleted these two replicates and performed a metabolome differential analysis. A total of 6455 metabolites were identified in the four *C. oleifera* groups (SP48, SP72, CP48 and CP72). We detected a total of 60 differentially expressed metabolites (DEMs) in SP48_vs_SP72, CP48_vs_CP72, SP48_vs_CP48 and SP72_vs_CP72 (Table S7). The numbers of DEMs for each comparison were 33, 31, 28 and 18, including four, seven, three and three up-regulated metabolites and 29, 24, 25 and 15 downregulated metabolites, respectively (Figure 7B). Venn diagrams showed that there were 43 DEMs in self- and cross-pollinated pistils at 48 h and 72 h (Figure 7A).

Cluster analysis revealed that 43 DEMs could be clustered into five categories (I–Ⅴ). The 27 DEMs in Clusters I–III had relatively high production in SP (Figure 7C). Among them, five metabolites (jasmine lactone, maltotriose, raffinose, d-maltose and matairesinol) were highly synthesized in self-pollinated pistils at both 48 h and 72 h, while 17 metabolites (1-aminocyclopropanecarboxylic acid, 2-ethoxyethanol, diethanolamine, hypoxanthine, 2-hydroxyadenine, adenine, uracil, guanosine, uridine, l-histidine, lysoPC (16:0), gamma-butyrolactone, 4-aminobutyric acid, l-glutamine, adenosine, l-glutamate and 2-pyrrolidineacetic acid) had significantly higher production at SP48 combinations than other groups. Moreover, five metabolites (d-mannitol, phenylethylamine, 3-alpha-mannobiose, maltopentaose and myo-Inositol) were significantly synthesized at SP72. There were 15 DEMs in Clusters IV–V with higher production in CP. Six metabolites in CP48 (quinate, linoleic acid, phloetin, procyanidin B2, uridine and 5′-diphosphate (UDP)) and nine metabolites in CP72 (d-proline, MG (16:0), lanosterol, l-leucine, l-valine, PC (16:0/16:0), erucamide, l-asparagine and l-isoleucine) had significant higher production than other groups.

KEGG pathway enrichment analysis showed that there were 16 DEMs linked to ABC transporters, which are also related to SI in plants [52]. Thirteen of them (l-aspartate, l-phenylalanine, choline, l-histidine, myo-inositol, l-glutamine, sucrose, l-glutamate, maltotriose, d-maltose, raffinose, d-mannose and d-mannitol) had higher production in SP, while three of them (l-isoleucine, l-valine and l-leucine) were significantly synthesized at CP72 (Table S7). These DEMs and related metabolic pathways in self- and cross-pollinated pistils might be part of the LSI reactions in *C. Oleifera*.

### 2.8. Integrated Transcriptomic, Proteomic and Metabolic Datasets

There were 51 metabolic pathways which were shared by at least two DETs, DEPs and DEMs in self- and cross-pollinated pistils from the transcriptome, proteome and metabolome datasets (Figure S5 and Table S8). Twenty-three metabolic pathways appeared associated to the three sets, such as fatty acid biosynthesis, aminoacyl-tRNA biosynthesis, alanine, aspartate and glutamate metabolism, arginine and proline metabolism, arginine biosynthesis, β-alanine metabolism, phenylalanine metabolism, pyrimidine metabolism and valine, and leucine and isoleucine degradation. Likewise, 19 additional pathways were found in common between DETs and DEPs (Figure S5 and Table S8), such as plant MAPK signaling pathways, plant hormone signal transduction, RNA degradation, ubiquitin-mediated proteolysis and oxidative phosphorylation, etc. Moreover, another seven were additionally shared by DETs and DEMs, such as starch and sucrose metabolism, and ABC transporters, while only two metabolic pathways (cAMP signaling pathway and valine, leucine and isoleucine biosynthesis) were indicated by DEPs and DEMs.

**Figure 8:**
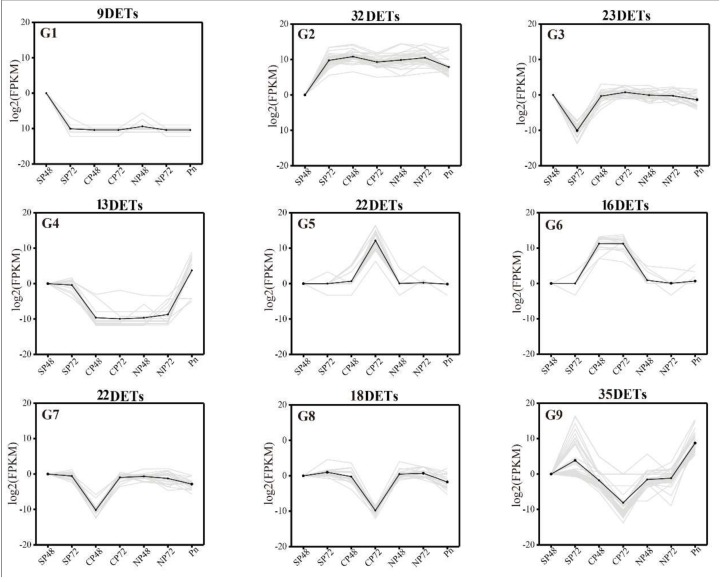
Trend analysis of differentially expressed genes showing the transcriptome expression profiles. Nine clusters were identified. SP48/72, self-pollinated pistils at 48 h/72 h; CP48/72, cross-pollinated pistils at 48 h/72 h; NP48/72, non-pollinated pistils at 48 h/72 h and Pn, mature pollen. FPKM = fragments per kilobase per million mapped fragments.

To further understand the relationship between DETs expression and the amounts of DEPs and DEMs, we analyzed the correlation between them using Pearson’s correlation statistics (Table S9). First, DETs with the same expression pattern could be found by trend analysis, as shown in Figure 8 Nine trends (G1–G9) were found, with *p* < 0.05. In the nine trend graphs, 190 transcripts that responded strongly to self- and cross-pollination reactions associated with 226 proteins and 38 metabolites (Table S9). Among them, the expression level of G1 in SP48 was relatively higher than other groups, and four DEPs and 16 DEMs had the same trend of synthesis. Conversely, 32 DETs in G2 had lower expression in SP48 than other groups and related to one DEP and three DEMs. G3 and G7 were expressed at the lowest in SP72 and CP48, respectively. Thirteen DETs in G4 were abundantly expressed in SP48, SP72 and mature pollen, while G6 were abundantly expressed in CP. The expression trends of G5 and G8 were opposite. The former was significantly higher in CP72 than other groups, and the latter was significantly lower in CP72 than other groups. Interestingly, in G9, 35 DETs, 32 DEPs and 5 DEMs had the highest expression in SP72, but the expression levels in CP72 were the lowest. These screened transcripts, proteins and metabolites could be used as the basis for studying the molecular mechanism of LSI in *C. oleifera*.

## 3. Discussion

*C. oleifera* seeds are the source of healthy edible oil. However, the seed yield is low due to low natural seeding rates caused by the LSI of oil-tea tree [28,53,54]. The current study observed the difference of pollen tubes in the pistils between self- and cross-pollinated pistils by cytological observation. We found that the pollen tube would grow to the upper part of the ovary at the base of the style and then showed abnormalities, such as swelling, bifurcation and wavy, and stopped growing between 48 h and 72 h after self-pollination. The cross-pollinated pollen tubes entered the ovary at 48 h and entered the embryo sac through the micropyle at 72 h. It has been reported that the inhibition of pollen tubes in the self-pollinated oil-tea tree belonged to a type of PCD [33]. All these findings confirmed that the SI of *C. oleifera* was the LSI type by PCD. Furthermore, the current study used transcriptomics, proteomic and metabolomic technologies to study the molecular level differences between self- and cross-pollinated pistils in *C. oleifera*. Data analysis revealed that a large regulatory network existed in the LSI process with PCD in self-pollinated pollen tubes. The network included the transcripts, protein products and metabolites associated with plant MAPK signaling pathways, plant hormone signal transduction, ABC transporters and ubiquitin-mediated proteolysis. The relationship among PCD, metabolic pathways and SI are described in details as follows.

### 3.1. PCD-Regulated Genes Regulate the Inhibition of Pollen Tube in Self-Pollination

It has been reported that PCD specifically occurred in incompatible pollen tubes of *Pyrus pyrifolia* [21,34]. Chen [55] speculated that after the self-pollination signal was recognized, it was amplified by a cascade system, which changes the function of cell metabolism and eventually leads to PCD in the pollen tube. Transmission and scanning electron microscopy showed that the death of the *C. oleifera* pollen tube in the self-pollinated pistils belonged to PCD [33].

PCD could be controlled by both positive and negative regulators. In this study, 20 putative key PCD-related genes were detected based on their expression patterns, which included 10 positive genes (*ADHⅢ, AMC4, CAT2*, *CRY1*, *RING1*, *RSP, POB1, SRC2*, *UBA2C* and *XCP1*) and 10 negative genes (*ACA4*, *BON3*, *CBSX5, CNX1, CRLK2, IMPA4, P2C63, RBOHA* and *VPS45*) [46,47,48,49,50,51] (Table S5). Most of the positive genes exhibited relatively high levels of expression in SP by Duncan’s test, such as *CAT2*, *CRY1*, *RING1*, *RSP* and *UBA2C* (Figure 5A). Relatively, the negative genes showed low expression levels in SP and high expression in cross- or non-pollinated pistils, such as *ACA4*, *BON3*, *CBSX5* and *RBOHA* (Figure 5A). It has been reported that S-RNase could specifically cause degradation of RNA in the microfilament skeleton of pollen tubes, which causes the occurrence of pollen tube PCD in *Pyrus pyrifolia* [21,56]. We also found that six DETs and one DEP were associated with RNA degradation (Tables S4 and S6), which could trigger a signaling cascade resulting in PCD culminated by factors that determine style incompatibility [57,58]. The results suggested the important role of PCD-related genes and protein produced in the inhibition of the pollen tube growth of LSI in *C. Oleifera*.

### 3.2. MAPK Signaling Pathway Involvement in SI of C. Oleifera

MAPKs were known to be functionally involved in the activation of defense and stress responses [43,59,60]. It has been reported that SI and the plant innate immunity systems have common pathways [23,61]. SI inhibition of pollen tube growth could be regarded as a stress response, so it was speculated that MAPKs might be involved in the SI response [62]. A MAPK kinase p56 was implicated in the SI-induced signaling cascade in Papaver pollens, which was specifically expressed in incompatible pollen [42]. 

In this study, we found that 16 DETs and two DEPs were associated with the MAPK signaling pathway. Among them, *ETR*, *CTR1*, *EIN2*, *EIN3* and *EBF1/2*-encoding proteins that participate in plant hormone signal transduction (Figure 4). *CaM4* and *WRKY33* were associated with MAPK signal transduction, and their expression levels in CP72 were 23.7 and 8.7 times higher than that in SP72, respectively (Table S3). Studies have shown that pollen tube growth requires a Ca^2+^ gradient at the tip. SI makes the extracellular calcium influx eliminating pollen tube tip Ca^2+^ gradient and inhibits the pollen tube growth [63]. As a transcription factor, *WRKYs* can act as positive or negative regulators in a defense response [64]. We speculate that *CaM* and *WRKY33* could respond to LSI and might play a negative regulatory role in the PCD process of self-pollinated pollen tubes in C. oleifera. 

### 3.3. Plant Hormone Signal Transduction Involvement in SI of C. Oleifera

Plant hormones play an important regulatory role in plant PCD. Studies have shown that the PCD process in the development of maize endosperm required a balance between ABA and ethylene [65,66,67]. This suggested that plant hormones might work together to regulate plant cell PCD. DETs after self- and cross-pollination were significantly enriched in plant hormone signal transduction pathway and plant–pathogen interaction pathway using transcriptomics in pears [22]. Likewise, it also demonstrated that jasmonate (JA) and abscisic acid (ABA) might enhance the expression level of *S-RNase*, which caused the PCD of the pollen tube in SI. It has been reported that the entries of both self and non-self S-RNase into pollen tubes of apples (*Malus domestica*) stimulated JA production, in turn inducing the accumulation of *MdMYC2* transcripts, a transcription factor in the JA signaling pathway widely considered to be involved in plant defense processes [23].

In this study, we found that there were 16 DETs encoding JA and ETH signaling-related components and one DEP involved in plant hormone signal transduction. Among them, *COI1.2* and *COI1.3* were significantly higher expressed at SP72 than in the other groups (Figure 4), and the expression level in pollen was the lowest. COI1 is an F-BOX domain protein that forms a complex with SCF, which is involved in JA-regulated plant defense responses to biotic stress [68]. The SCF complex selectively degrades non-self S-RNase in the SI reaction by a ubiquitin-mediated protease degradation system, thereby causing the pollen tube PCD [69,70].

### 3.4. ABC Transporters Involvement in SI of C. Oleifera

ABC transporters are a large family of transmembrane transporters, also known as ATP-binding cassette transporters, and are currently the largest and most versatile family of proteins [71]. It has reported that MdABCF assisted in transportation of either self or non-self S-RNase into the pollen tube. Moreover, MdABCF coordinated with the cytoskeleton to transport S-RNase [52]. In our study, it was found that six DETs-encoded proteins and 16 DEMs in SP and CP are related to ABC transporters (Tables S4 and S7). The DETs included *ABCB* (cb1496_c2/f1p0/2420, cb1707_c15/f1p0/2047, cb1707_c16/f1p0/3057), *ABCC* (cb485_c32/f1p0/4653, cb485_c8/f1p0/2223) and *ABCG* (cb8540_c10/f1p2/2041), with higher expression at SP48 than CP48. DEMs included metabolites such as l-aspartate, choline, sucrose and maltotriose, which had higher production in SP. Other studies have shown that ABC transporters were closely related to transportation of plant hormones [72], which were closely related to plant SI. ABC transports might be involved in the regulatory network of LSI in *C. oleifera.*

### 3.5. Ubiquitin-Mediated Proteolysis Involvement in SI of C. Oleifera

It has been reported that incompatible pollen *SCR* could activate *SRK* in a S-haploid-specific manner in the SSI plants [73]. This process leads to an intracellular signal transduction cascade, ensuing downstream reactions with an E3 ubiquitin ligase [74], which is associated with ubiquitin-mediated proteolysis. In GSI plants, S-RNase could be specifically prevented from being degraded by ubiquitin 26S proteasome after self-pollination, retaining its activity as a cytotoxin to degrade its own pollen tube tRNA, thereby inhibiting the pollen tube growth and leading to the occurrence of the SI reaction [69,70,75,76]. We found the DETs *JAZ* and *EIN3* directly affected the third metabolic pathway ubiquitin-mediated proteolysis, including three genes *SIAH1*, *RCHY1* and *TRIP12*, which encode for three types of the E3 ubiquitin ligase (Figure 4) [77]. SIAH1, RCHY1 and TRIP12 were reported to play important roles in plant salt tolerance, drought, high temperature, low temperature stress and ABA signaling pathways. Further studies showed that ubiquitin ligase reduced reactive oxygen species (ROS) production and cell death, thereby positively regulating the salt stress response [78]. In recent years, more and more studies have found that SI could cause plant immune responses. For example, increased PA levels in pollen tubes initially played a protective role in incompatible pollen, until sustained PbrS-RNase activity reaches the point of no return and pollen tube growth ceases in pears [21]. A gamma-thionin protein from apples, MdD1, was required for defense against the S-RNase-induced inhibition of the pollen tube prior to self/non-self recognition [23]. Our results showed that the ubiquitin-mediated proteolysis pathway might directly be involved in the LSI or could assume a self-defense role in the LSI process of *C. oleifera*.

## 4. Materials and Methods

### 4.1. Plant Materials

Two oil-tea tree varieties, “Hua Xin” and “Hua Jin”, developed by the Central South University of Forestry and Science and widely cultivated in the Hunan Province of China were selected for the study. These two varieties bloomed from October to November each year, and fruits ripened in late October of the following year. The experimental materials were collected from an orchard in Wangcheng District, Changsha City, Hunan Province (latitude 28°05′ N, longitude 113°21′ E).

### 4.2. Pollination Treatment, Sample Collection and Cytological Observation

Three pollination treatments were designed, including (1) self-pollination (SP): “Hua Xin” × “Hua Xin”, (2) cross-pollination (CP): “Hua Xin” × “Hua Jin” and (3) non-pollination (NP): pistil of “Hua Xin” after emasculation. Mature pollens of “Hua Xin” and “Hua Jin” were collected from anthers in advance during the bud stage and placed in sulfate paper bags and kept at 25 °C for 8 h. Mature pollen (Pn) samples of “Hua Xin” (6 g) were stored in liquid nitrogen for RNA-seq. Pollination treatments were conducted in the field between 9:00–11:00 AM or 1:00–3:00 PM on sunny days in October 2017. For SP and CP, the flowers were emasculated and pollinated. The stigmas were not stained by any pollen until they became receptive. After pollination treatments, all the pistils were protected by sulfate paper bags. At 48 h and 72 h after pollination, pistils were collected and stored at −80 °C. There were 7 groups of samples (SP48, SP72, CP48, CP72, NP48, NP72 and Pn) for transcriptome, with 3 biological replicates of each group. Four groups of samples (SP48, SP72, CP48 and CP72) were used for proteome and metabolome, with 3 and 6 biological replicates, respectively (Table S1). Each replicate contained 60 pistils. In addition, 120 pistils of each treatment were used for cytological observation with the paraffin sectioning method [53] and quantitative real-time PCR analysis.

### 4.3. RNA Preparation

Total RNA was extracted from all the samples by grinding the tissues in TRIzol reagent (Life technologies, California, CA, USA). RNA purity (OD_260/280_) was measured using Nanodrop ND200 (Thermo Fisher, Waltham, MA, USA). RNA concentration, RIN (RNA integrity number) value and 28S/18S value were measured using Agilent 2100 (Agilent, California, CA, USA). The total RNA with RIN ≥ 8, 28S/18S ≥ 1.5 and OD_260/280_ ≥ 1.8 of samples were used for deep sequencing. 

### 4.4. PacBio cDNA Library Construction and Third-Generation Sequencing

Total RNA was extracted one by one from 21 samples (Table S1). Ten microliters of total RNA from each sample were pooled. The pooled RNA was reverse-transcribed into cDNA using Clontech SMARTer PCR cDNA Synthesis Kit (Takara, Dalian, China) according to the protocol provided by the manufacturer for third-generation sequencing. The SMRTbell template was annealed to the sequencing primer and bound to polymerase and sequenced on the PacBio RS II platform to obtain a representative full-length transcriptome for *C. oleifera* [79]. Proofreading error correction software was used for large-scale high-accuracy PacBio correction through iterative short-read consensus by second-generation sequencing [39]. The final transcriptome isoform sequences were filtered by removing the redundant sequences with software CD-HIT-v4.6.7 [80] using a threshold of 0.99 identities. Basic isoform annotations were blast-searched by Diamond software (Version 3.1.4) [81] against NCBI non-redundant protein sequences (NR) (May 21, 2018), gene ontology (GO) (May 21, 2018) [82], euKaryotic Ortholog Groups (KOG) (May 21, 2018) [83], Kyoto Encyclopedia of Genes and Genomes (KEGG) (May 21, 2018) [84] and Swiss-Prot Protein Sequence Database (Swiss-Prot) (May 21, 2018) [85]. The open reading frames (ORFs) were detected using the ANGLE software [86] for transcript sequences to obtain the coding sequences (CDS), protein sequences and UTR sequences.

### 4.5. Illumina cDNA Library Construction and Second-Generation Sequencing

After total RNA was extracted, eukaryotic mRNA was enriched by Oligo [87] beads, while prokaryotic mRNA was enriched by removing rRNA using Ribo-ZeroTM Magnetic Kit (Epicentre, New York, NY, USA city, state abbrev., country). RNA was sequenced using Illumina HiSeqTM2500 by Gene De novo Biotechnology Co (Wuhan, China) [88]. The rRNA-removed reads of each sample were then mapped to redundancy isoform from ISO-seq by TopHat (version 2.0.3.12) [89]. To identify DETs with a fold change ≥ 2 and a false discovery rate (FDR) < 0.01 of 8 sample pairs (SP48_vs_SP72, CP48_vs_CP72, SP48_vs_CP48, SP72_vs_CP72, SP48_vs_NP48, CP48_vs_NP48, SP72_vs_NP72 and CP72_vs_NP72), the DESeq2 package of the R Project was used [90]. 

### 4.6. Proteomic Analysis 

In this experiment, proteomic changes were performed by isobaric tags for relative and absolute quantitation (iTRAQ) [91]. The pistil samples of the different pollination treatments were extracted by SDT lysis [92], and then the protein content was quantified by the BCA method. The appropriate amount of proteins in each sample were trypsinized by the Filter Aid Proteome preparation (FASP) method [92]. The hydrolyzed peptides were desalted by C18 cartridge and lyophilized and reconstituted with 40 μL dissolution buffer. The peptides (100 μg) of each sample were labeled according to the AB SCIEX iTRAQ Labeling Kit instructions [91]. Each set of labeled peptides was mixed and fractionated using AKTA Purifier 100. Each fractionated sample was separated using a HPLC liquid phase system Easy nLC at 300 nL/min. The sample was chromatographed and subjected to mass spectrometry using a Q-Exactive mass spectrometer. Protein database was translated from the ISO-seq database. The library Mascot (Version 2.2) and Proteome Discoverer (Version 1.4) were used for identification and quantitative analysis with FDR < 0.01 using the RAW files of mass spectrometry. Principal component analysis (PCA) provides the evaluation of the reliability of experimental results, as well as operational stability. GOs’ annotation was performed on the target protein collection using Blast2GO [93], and the KEGG pathway annotation was performed on the target protein collection using KAAS (KEGG Automatic Annotation Server) software [94]. The DEPs were defined according to the standard of expression fold changed more than 1.2 times and *p-*value < 0.05.

### 4.7. Metabolomic Analysis

Metabolomics methods based on HILIC and RPLC UHPLC-Q-TOF MS techniques were used [95]. The XCMS program (http://enigma.lbl.gov/xcms-online/) was used for peak alignment, retention time correction and peak area extraction. Metabolite structure identification was performed using a method of accurate mass-matching (<25 ppm) and secondary spectral matching to retrieve a self-built database [96,97]. For the data extracted by XCMS, ion peaks with missing values >50% in the group were removed. SIMCA-P 14.1 (Umetrics, Umea, Sweden) was used for pattern recognition, and the data was preprocessed by Pareto-scaling. Multidimensional statistical analysis, including unsupervised PCA analysis, supervised partial least squares discriminant analysis (PLS-DA) and orthogonal partial least squares discriminant analysis (OPLS-DA), were performed. DEMs were identified using VIP (variable importance for the projection) >1 and *p-*value < 0.1 as screening criteria.

### 4.8. Transcript, Protein and Metabolite Correlation Analysis

Through the statistics of the data, the metabolic pathways in which DETs, DEPs and DEMs were detected in self- and cross-pollinated pistils in *C. oleifera* were analyzed. To compare the concordance among transcriptome, proteome and metabolome changes, trend and correlation analysis were performed on DETs (Figure S4), DEPs (Figure S6) and DEMs (Figure S7). First, DETs with the same expression pattern could be found by trend analysis with online software (https://www.omicshare.com/tools/Home/Soft/trend). Nine trends were selected with *p* < 0.05, in which the transcripts expression trends were closely related to LSI in *C. oleifera*. The Cor program in R Project [98] was used to calculate the Pearson’s correlation coefficient of DETs, DEPs and DEMs. With the Pearson’s correlation coefficient greater than 0.8 at the threshold, DEPs and DEMs with the same expression pattern of DETs were screened out.

### 4.9. Quantitative RT-PCR

Total RNA was extracted using plant RNA kit (OMEGA, Norcross, GA, city, state abbrev., USA) according to manufacturer’s instructions, while RNase-free DNase I (Fermatas, Toronto city, Canada) was used to eliminate the remaining potential genomic DNA. The total RNA was reverse-transcribed into the first single-stranded cDNA by the PrimeScript^TM^ RT reagent kit with gDNA Eraser (TaKaRa, Japan). All quantitative RT-PCR (qRT-PCR) were performed with the CFX96^TM^ real-time PCR system (BIO-RAD, Hercules, CA, city, state abbrev., USA) by SYBR Premix ExTaqTM (2×) (TaKaRa, Japan). *CoGAPDH* was used as the reference gene for *C. oleifera* [99]. The reactions were carried out in triplets using independent biological samples. The primers used were listed in Table S10. The mRNA expression levels were analyzed by Software Bio-Rad CFX Manager with 2^−ΔΔCT^ method [100].

### 4.10. Statistical Analysis

All experimental data were expressed as the mean of three or six independent biological repeats and the standard deviation (mean ± SD). Statistical analyses were performed by SPSS software using the Duncan’s test.

### 4.11. Availability of Sequence Data

The PacBio SMRT reads and the Illumina SGS reads generated in this study have been deposited in the NCBI Sequence Read Archive (http://www.ncbi.nlm.nih.gov/sra). The BioProject are PRJNA606665 and PRJNA606862, respectively. The mass spectrometry proteomics data by iTRAQ have been deposited to the ProteomeXchange Datasets (http://www.proteomexchange.org) with the submission reference 1-2020015-117203.

## Supplementary Materials

Supplementary materials can be found at https://www.mdpi.com/1422-0067/21/5/1600/s1. Figure S1. The classification statistics of reads of insert, functional annotation and correlation map of *C. oleifera* full-length transcriptome. (A) Proportion of reads of insert. (B) Venn diagram of NR, COG, KEGG, SwissProt and InterPro results for *C. oleifera* transcriptome. (C) Gene ontology enrichment analysis of *C. oleifera* PacBio transcript isoforms. (D) KEGG metabolic pathway classification of *C. oleifera* PacBio transcript isoforms. (E) KOG classification of *C. oleifera* PacBio transcripts. (F) Principal component analysis (PCA) of the oil-tea camellia transcriptome of 21 independent samples collected from SP48/72, CP48/72, NP48/72 and Pn. SP48/72, self-pollinated pistils at 48 h/72 h; CP48/72, cross-pollinated pistils at 48 h/72 h; NP48/72, non-pollinated pistils at 48 h/72 h and Pn, mature pollen. Figure S2. Comparison of transcriptome results from Illumina and PacBio-sequencing platforms. (A) Transcript average length, mean length, N50; (B,E) percentages of functional annotation; (C,G) coding sequence predictions; (D,F) percentages of NR, GO, KEGG, KOG and SwissProt annotation (A–D) and Illumina (A,E–G)-sequencing platforms. Figure S3. Principal component analysis (PCA) of the oil-tea camellia proteome of 12 independent samples collected from self- and cross-pollinated pistils at 48 and 72 hours after pollination. SP48/72, self-pollinated pistils at 48 h/72 h and CP48/72, cross-pollinated pistils at 48 h/72 h. Figure S4. Principal component analysis (PCA) of the oil-tea camellia metabolome of 24 independent samples collected from self- and cross-pollinated pistils at 48 and 72 hours after pollination. SP48/72, self-pollinated pistils at 48 h/72 h and CP48/72, cross-pollinated pistils at 48 h/72 h. Figure S5. The Venn diagram of the common metabolic pathways shared by DETs, DEPs and DEMs in self- and cross-pollinated pistils at 48 and 72 hours after pollination. Table S1. The sample names of transcriptome, proteome, and metabolome. Table S2. Quality testing result of total RNA in the transcriptome of 21 independent samples collected from SP48/72, CP48/72, NP48/72 and Pn. SP48/72, self-pollinated pistils at 48 h/72 h; CP48/72, cross-pollinated pistils at 48 h/72 h; NP48/72, non-pollinated pistils at 48 h/72 h and Pn, mature pollen. Table S3. The summary of polymerase reads in SMRT-based RNA-sequencing. Table S4. Expression and annotation of DETS in SP48_vs_CP48 and SP72_vs_CP72. SP48/72, self-pollinated pistils at 48 h/72 h and CP48/72, cross-pollinated pistils at 48 h/72 h. Table S5. Expression pattern of PCD-related genes in 7 groups (SP48, SP72, CP48, CP72, NP48, NP72 and Pn). SP48/72, self-pollinated pistils at 48 h/72 h; CP48/72, cross-pollinated pistils at 48 h/72 h; NP48/72, non-pollinated pistils at 48 h/72 h and Pn, mature pollen. Table S6. List of DEPs in self- and cross-pollinated pistils at 48 and 72 hours after pollination. Table S7. List of DEMs in self- and cross-pollinated pistils at 48 and 72 hours after pollination. Table S8. The common metabolic pathways shared by DETs, DEPs and DEMs in self- and cross-pollinated pistils at 48 and 72 hours after pollination. Table S9. The statistics of correlation between DETs, DEPs and DEMs. Table S10. Primers of qRT-PCR.

## Abbreviations

COI1	coronatine-insensitive protein 1 (K13463)
JAZ	jasmonate-zim-domain protein (K13464)
ETR	ethylene receptor (K14509)
CTR1	serine/threonine-protein kinase 1 (K08856)
EIN2	ethylene-insensitive protein 2 (K14513)
EIN3	EIN3-like protein (K14514)
EBF1/2	EIN3-binding F-box protein (K14515)
CALM	calmodulin (K02183)
XRN4	5′-3′ exoribonuclease 4 (K20553)
SIAH1	E3 ubiquitin-protein ligase SIAH1 (K04506)
RCHY1	RING finger and CHY zinc finger domain-containing protein 1 (K10144)
TRIP12	E3 ubiquitin-protein ligase UPL4 (K10590)
WRKY33	WRKY transcription factor 33 (K13424)
ARR-B	two-component response regulator ARR2 isoform (K14491)
ABA2	xanthoxin dehydrogenase (EC:1.1.1.288)
TRA2	transformer-2 protein
TUBA	tubulin alpha
TGA	transcription factor TGA
PLCD	phosphatidylinositol phospholipase C, delta (EC:3.1.4.11)
PFN	profilin
MAPK1_3	mitogen-activated protein kinase 1/3 (EC:2.7.11.24)
SF3B4	splicing factor 3B subunit 4
EIF4A	translation initiation factor 4A
UBE1	ubiquitin-activating enzyme E1 (EC:6.2.1.45)
WDR61	WD repeat-containing protein 61

## References

[1] Zhuang R.L. (2008). Comprehensive Utilization of Tea-Oil Fruits, Tea-Oil Tree (Camellia oleifera Abel.) of China.

[2] Xiao Y. (2006). Investigation and prospect of bio-active components in vegetable oil. Cereal Food Ind..

[3] Li H., Fang X.Z., Zhong H.Y., Fei X.Q., Luo F. (2014). Variation of physicochemical properties and nutritional components of oil-tea Camellia seeds during riping. For. Res..

[4] Li H., Zhou G., Zhang H., He Y. (2010). Chemical constituents and biological activities of saponin from the seed of *Camellia oleifera*. Sci. Res. Essays.

[5] Goldberg E.E., Kohn J.R., Lande R., Robertson K.A., Smith S.A., Igić B. (2010). Species selection maintains self-incompatibility. Science.

[6] Franklin-Tong N., Franklin F.C.H. (2003). Gametophytic self-incompatibility inhibits pollen tube growth using different mechanisms. Trends Plant Sci..

[7] Waligórski P., Szaleniec M. (2010). Prediction of white cabbage (*Brassica oleracea var. capitata*) self-incompatibility based on neural network and discriminant analysis of complex electrophoretic patterns. Comput. Biol. Chem..

[8] Gibbs P.E. (2014). Late-acting self-incompatibility—The pariah breeding system in flowering plants. New Phytol..

[9] Shi G.J., Hou X.L. (2004). Measurement of Self-incompatible by Fluoroscope Observation in Non-heading Chinese Cabbage. J. Wuhan Bot. Res..

[10] Xu Y., Zhang S. (2003). Characterization and molecular mechanism of gametophytic self-incompatibility in pears. J. Fruit Sci..

[11] Li X., Zhang S., Wu J., Wu H. (2006). Studies on S genotypes and molecular mechanism of self-incompatibility in cherry. Biotechnol. Bull..

[12] Stein J.C., Howlett B., Boyes D.C., Nasrallah M.E., Nasrallah J.B. (1991). Molecular cloning of a putative receptor protein kinase gene encoded at the self-incompatibility locus of *Brassica oleracea*. Proc. Natl. Acad. Sci. USA.

[13] Watanabe M., Suzuki G., Takayama S. (2008). Milestones Identifying Self-Incompatibility Genes in *Brassica* Species: From Old Stories to New Findings. Self-Incompat. Flower. Plants.

[14] Schopfer C.R., Nasrallah M.E., Nasrallah J.B. (1999). The male determinant of self-incompatibility in *Brassica*. Science.

[15] Takasaki T., Hatakeyama K., Suzuki G., Watanabe M., Isogai A., Hinata K. (2000). The S receptor kinase determines self-incompatibility in *Brassica stigma*. Nature.

[16] Kohji M., Hiroshi S., Megumi I., Fang-Sik C., Masao W., Akira I., Seiji T. (2004). A membrane-anchored protein kinase involved in *Brassica* self-incompatibility signaling. Science.

[17] Kakita M., Murase K., Iwano M., Matsumoto T., Watanabe M., Shiba H., Isogai A., Takayama S. (2007). Two distinct forms of M-locus protein kinase localize to the plasma membrane and interact directly with S-locus receptor kinase to transduce self-incompatibility signaling in *Brassica rapa*. Plant Cell.

[18] Murfett J., Atherton T.L., Mou B., Gassert C.S., McClure B.A. (1994). S-RNase expressed in transgenic *Nicotiana* causes S-allele-specific pollen rejection. Nature.

[19] Sijacic P., Wang X., Skirpan A.L., Wang Y., Dowd P.E., McCubbin A.G., Huang S., Kao T.H. (2004). Identification of the pollen determinant of S-RNase-mediated self-incompatibility. Nature.

[20] McClure B. (2006). New views of S-RNase-based self-incompatibility. Curr. Opin. Plant Biol..

[21] Chen J., Wang P., Graaf B.H.J.D., Zhang H., Wu J. (2018). Phosphatidic Acid Counteracts S-RNase Signaling in Pollen by Stabilizing the Actin Cytoskeleton. Plant Cell.

[22] Shi D.Q., Tang C., Wang R.Z., Gu C., Wu X., Hu S., Jiao J., Zhang S.L. (2017). Transcriptome and phytohormone analysis reveals a comprehensive phytohormone and pathogen defence response in pear self-/cross-pollination. Plant Cell Rep..

[23] Gu Z.Y., Li W., Doughty J., Meng D., Yang Q., Yuan H., Li Y., Chen Q.J., Yu J., Liu C.S. (2019). A gamma-thionin protein from apple, MdD1, is required for defense against S-RNase-induced inhibition of pollen tube prior to self/non-self recognition. Plant Biotechnol. J..

[24] Cope F.W. (1962). The mechanism of pollen incompatibility in *Theobroma cacao L*. Heredity (Edinb.).

[25] Ford C.S., Wilkinson M.J. (2012). Confocal observations of late-acting self-incompatibility in *Theobroma Cacao L*. Sex. Plant Reprod..

[26] Kenrick J., Kaul V., Williams E.G. (1986). Self-incompatibility in *Acacia retinodes*: Site of pollen-tube arrest is the nucellus. Planta.

[27] Chen X., Hao S., Wang L., Fang W., Wang Y., Li X. (2012). Late-acting self-incompatibility in tea plant (*Camellia sinensis*). Biologia.

[28] Chao G., Yuan D., Yang Y., Wang B., Liu D., Feng Z., Tan X. (2015). Anatomical Characteristics of Self-Incompatibility in *Camellia oleifera*. Sci. Silvae Sin..

[29] Zhou Q., Zheng Y. (2015). ComparativeDe NovoTranscriptome Analysis of Fertilized Ovules in *Xanthoceras sorbifolium* Uncovered a Pool of Genes Expressed Specifically or Preferentially in the Selfed Ovule That Are Potentially Involved in Late-Acting Self-Incompatibility. PLoS ONE.

[30] Zhang C.C., Wang L.Y., Wei K., Wu L.Y., Li H.L., Zhang F., Cheng H., Ni D.J. (2016). Transcriptome analysis reveals self-incompatibility in the tea plant (*Camellia sinensis*) might be under gametophytic control. Bmc Genom..

[31] Zhang C.C., Tan L.Q., Wang L.Y., Wei K., Wu L.Y., Zhang F., Cheng H., Ni D.J. (2016). Cloning and characterization of an S-RNase gene in *Camellia sinensis*. Sci. Hortic..

[32] Lanaud C., Fouet O., Legavre T., Lopes U., Sounigo O., Eyango M.C., Mermaz B., Da Silva M.R., Loor Solorzano R.G., Argout X. (2017). Deciphering the Theobroma cacao self-incompatibility system: From genomics to diagnostic markers for self-compatibility. J. Exp. Bot..

[33] Gao C. (2017). The Cytological Study on Late-acting Self-incompatibility in *Camellia oleifera*. PhD Thesis.

[34] Wang C.L., Xu G.H., Jiang X.T., Gong C., Zhang S.L. (2009). S-RNase triggers mitochondrial alteration and DNA degradation in the incompatible pollen tube of *Pyrus pyrifolia* in vitro. Plant J..

[35] Martin J.A., Wang Z. (2011). Next-generation transcriptome assembly. Nat. Rev. Genet..

[36] Sharon D., Tilgner H., Grubert F., Snyder M. (2013). A single-molecule long-read survey of the human transcriptome. Nat. Biotechnol..

[37] Minoche A.E., Dohm J.C., Schneider J., Holtgräwe D., Viehöver P., Montfort M., Rosleff Sörensen T., Weisshaar B., Himmelbauer H. (2015). Exploiting single-molecule transcript sequencing for eukaryotic gene prediction. Genome Biol..

[38] Koren S., Schatz M.C., Walenz B.P., Martin J., Howard J.T., Ganapathy G., Wang Z., Rasko D.A., McCombie W.R., Jarvis E.D. (2012). Hybrid error correction and de novo assembly of single-molecule sequencing reads. Nat. Biotechnol..

[39] Hackl T., Hedrich R., Schultz J., Förster F. (2014). *proovread*: Large-scale high-accuracy PacBio correction through iterative short read consensus. Bioinformatics.

[40] Li Q., Li Y., Song J., Xu H., Xu J., Zhu Y., Li X., Gao H., Dong L., Qian J. (2014). High-accuracyde novoassembly and SNP detection of chloroplast genomes using a SMRT circular consensus sequencing strategy. New Phytol..

[41] Šamaj J., Ovecka M., Hlavacka A., Lecourieux F., Meskiene I., Lichtscheidl I., Lenart P., Salaj J., Volkmann D., Bögre L. (2002). Involvement of the mitogen-activated protein kinase SIMK in regulation of root hair tip growth. Embo J..

[42] Li S., Frankling-Tong V. (2011). Modulating and Monitoring MAPK Activity During Programmed Cell Death in Pollen. Methods Mol. Biol..

[43] Ligterink W., Kroj T., Nieden U., Hirt H., Scheel D. (1997). Receptor-Mediated Activation of a MAP Kinase in Pathogen Defense of Plants. Science.

[44] Windels D., Bucher E. (2018). The 5′-3′ Exoribonuclease XRN4 Regulates Auxin Response via the Degradation of Auxin Receptor Transcripts. Genes.

[45] Vidhyasekaran P. Ubiquitin-Proteasome System-Mediated Protein Degradation in Defense Signaling. Signaling and Communication in Plants.

[46] Liu M., Li W., Zhao G., Fan X., Long H., Fan Y., Shi M., Tan X., Zhang L. (2019). New Insights of Salicylic Acid Into Stamen Abortion of Female Flowers in Tung Tree (*Vernicia fordii*). Front. Genet..

[47] Gao M.G.A., Gao M.G. (1999). Yariv reagent treatment induces programmed cell death in *Arabidopsis* cell cultures and implicates arabinogalactan protein involvement. Plant J..

[48] Li Y., Williams B., Dickman M. (2017). *Arabidopsis* B-cell lymphoma2 (Bcl-2)-associated athanogene 7 (BAG7)-mediated heat tolerance requires translocation, sumoylation and binding to WRKY29. New Phytol..

[49] Lu Z., Xu J., Li W., Zhang L., Cui J., He Q., Wang L., Jin B. (2017). Transcriptomic Analysis Reveals Mechanisms of Sterile and Fertile Flower Differentiation and Development in *Viburnum macrocephalum* f. keteleeri. Front. Plant Sci..

[50] Sala K., Malarz K., Barlow P.W., Kurczyńska E.U. (2017). Distribution of some pectic and arabinogalactan protein epitopes during *Solanum lycopersicum* (L.) adventitious root development. Bmc Plant Biol..

[51] Torres M.A., Dangl J.L., Jones J.D.G. (2002). Arabidopsis gp91phox homologues AtrbohD and AtrbohF are required for accumulation of reactive oxygen intermediates in the plant defense response. Proc. Natl. Acad. Sci. USA.

[52] Meng D., Gu Z., Li W., Wang A., Yuan H., Yang Q., Li T. (2014). Apple MdABCF assists in the transportation of S-RNase into pollen tubes. Plant J..

[53] Liao T., Yuan D.Y., Zou F., Gao C., Yang Y., Zhang L., Tan X.F. (2014). Self-sterility in *Camellia oleifera* may be due to the prezygotic late-acting self-incompatibility. PLoS ONE.

[54] Gao C., Yuan D., Yang Y., Wang B., Liu D., Zou F. (2015). Pollen tube growth and double fertilization in *Camellia oleifera*. J. Am. Soc. Hortic. Sci..

[55] X C. (2010). Identification of Self-incompatibility Model, cloning and expression of Correlative Gene in *Camellia sinensis*. Ph.D. Thesis.

[56] Liu Z.Q., Xu G.H., Zhang S.L. (2007). *Pyrus pyrifolia* stylar S-RNase induces alterations in the actin cytoskeleton in self-pollen and tubes in vitro. Protoplasma.

[57] Eaves D.J., Flores-Ortiz C., Haque T., Lin Z., Teng N., Franklin-Tong V.E. (2014). Self-incompatibility in *Papaver*: Advances in integrating the signalling network. Biochem. Soc. Trans..

[58] Wilkins K.A., Poulter N.S., Franklin-Tong V.E. (2014). Taking one for the team: Self-recognition and cell suicide in pollen. J. Exp. Bot..

[59] Yang K.Y., Liu Y., Zhang S. (2001). Activation of a mitogen-activated protein kinase pathway is involved in disease resistance in tobacco. Proc. Natl. Acad. Sci. USA.

[60] Kroj T., Rudd J.J., Nürnberger T., Gäbler Y., Lee J., Scheel D. (2003). Mitogen-activated protein kinases play an essential role in oxidative burst-independent expression of pathogenesis-related genes in parsley. J. Biol. Chem..

[61] Nasrallah J.B. (2005). Recognition and rejection of self in plant self-incompatibility: Comparisons to animal histocompatibility. Trends Immunol..

[62] Rudd J.J., Franklin-Tong V.E. (2003). Signals and targets of the self-incompatibility response in pollen of *Papaver rhoeas*. J. Exp. Bot..

[63] Franklin-Tong V.E., Holdaway-Clarke T.L., Straatman K.R., Kunkel J.G., Hepler P.K. (2002). Involvement of extracellular calcium influx in the self-incompatibility response of *Papaver rhoeas*. Plant J..

[64] Uluhan E., Keleş E.N., Tufan F. (2019). Analysis of WRKY Transcription Factors in Barley Cultivars Infected with *Fusarium culmorum*. Int. J. Life Sci..

[65] Young T.E., Gallie D.R. (2000). Regulation of programmed cell death in maize endosperm by abscisic acid. Plant Mol. Biol..

[66] Gunawardena A.H., Pearce D.M., Jackson M.B., Hawes C.R., Evans D.E. (2001). Characterisation of programmed cell death during aerenchyma formation induced by ethylene or hypoxia in roots of maize (*Zea mays* L.). Planta.

[67] Jong A.J.D., Yakimova E.T., Kapchina V.M., Woltering E.J. (2002). A critical role for ethylene in hydrogen peroxide release during programmed cell death in tomato suspension cells. Planta.

[68] Thatcher L.F., Manners J.M., Kazan K. (2009). Fusarium oxysporumhijacks COI1-mediated jasmonate signaling to promote disease development in *Arabidopsis*. Plant J..

[69] Daiki M., Hisayo Y., Kazuyuki A., Ryutaro T. (2012). Identification of a Skp1-like protein interacting with SFB, the pollen S determinant of the gametophytic self-incompatibility in *Prunus*. Plant Physiol..

[70] Chi X., Maofu L., Junkai W., Han G., Qun L., Yu’E Z., Jijie C., Tianzhong L., Yongbiao X. (2013). Identification of a canonical SCF(SLF) complex involved in S-RNase-based self-incompatibility of *Pyrus* (Rosaceae). Plant Mol. Biol..

[71] Martinoia E., Klein M., Geisler M., Bovet L., Forestier C., Kolukisaoglu Ü., Müller-Röber B., Schulz B. (2002). Multifunctionality of plant ABC transporters—More than just detoxifiers. Planta.

[72] Liu L., Zhao L., Chen P., Cai H., Hou Z., Jin X., Aslam M., Chai M., Lai L., He Q. (2020). ATP binding cassette transporters ABCG1 and ABCG16 affect reproductive development via auxin signaling in *Arabidopsis*. Plant J..

[73] Takayama S., Shimosato H., Shiba H., Funato M., Che F.-S., Watanabe M., Iwano M., Isogai A. (2001). Direct ligand–receptor complex interaction controls *Brassica* self-incompatibility. Nature.

[74] Haasen K.E., Goring D.R. (2010). The recognition and rejection of self-incompatible pollen in the Brassicaceae. Bot. Stud..

[75] Yuan H., Meng D., Gu Z., Li W., Wang A., Yang Q., Zhu Y., Li T. (2014). A novel gene, MdSSK1, as a component of the SCF complex rather than MdSBP1 can mediate the ubiquitination of S-RNase in apple. J. Exp. Bot..

[76] Dong M., Gu Z., Wang A., Hui Y., Wei L., Yang Q., Duan X., Li T. (2014). Screening and characterization of apple Rho-like GTPase (MdROPs) genes related to S-RNase mediated self-incompatibility. Plant Cell Tiss. Org..

[77] Jong-Jin P., Jakyung Y., Jinmi Y., Lae-Hyeon C., Jin P., Hee Joong J., Seok Keun C., Woo Taek K., Gynheung A. (2011). OsPUB15, an E3 ubiquitin ligase, functions to reduce cellular oxidative stress during seedling establishment. Plant J..

[78] Potocký M., Jones M., Bezvoda R., Smirnoff N., Žárský V. (2007). Reactive oxygen species produced by NADPH oxidase are involved in pollen tube growth. New Phytol..

[79] Liu X., Mei W., Soltis P.S., Soltis D.E., Barbazuk W.B. (2017). Detecting alternatively spliced transcript isoforms from single-molecule long-read sequences without a reference genome. Mol. Ecol. Resour..

[80] Li W., Godzik A. (2006). Cd-hit: A fast program for clustering and comparing large sets of protein or nucleotide sequences. Bioinformatics.

[81] Buchfink B., Xie C., Huson D.H. (2014). Fast and sensitive protein alignment using DIAMOND. Nat. Methods.

[82] Ashburner M., Ball C.A., Blake J.A., Botstein D., Cherry J.M. (2000). Gene ontology: Tool for the unification of biology. Nat. Genet..

[83] Tatusov R.L., Fedorova N.D., Jackson J.D., Jacobs A.R., Kiryutin B., Koonin E.V., Krylov D.M., Mazumder R., Mekhedov S.L., Nikolskaya A.N. (2003). The COG database: An updated version includes eukaryotes. Bmc Bioinform..

[84] Kanehisa M., Goto S., Kawashima S., Okuno Y., Hattori M. (2004). The KEGG resource for deciphering the genome. Nucleic Acids Res..

[85] Gasteiger E., Jung E., Bairoch A. (2001). SWISS-PROT: Connecting biomolecular knowledge via a protein database. Curr. Issues Mol. Biol..

[86] Shimizu K., Adachi J., Muraoka Y. (2006). ANGLE: A sequencing errors resistant program for predicting protein coding regions in unfinished cDNA. J. Bioinform. Comput. Biol..

[87] Fu H., Doelling J.H., Arendt C.S., Hochstrasser M., Vierstra R.D. (1998). Molecular organization of the 20S proteasome gene family from *Arabidopsis thaliana*. Genetics.

[88] Trapnell C., Williams B.A., Pertea G., Mortazavi A., Kwan G., van Baren M.J., Salzberg S.L., Wold B.J., Pachter L. (2010). Transcript assembly and quantification by RNA-Seq reveals unannotated transcripts and isoform switching during cell differentiation. Nat. Biotechnol..

[89] Kim D., Pertea G., Trapnell C., Pimentel H., Kelley R., Salzberg S.L. (2013). TopHat2: Accurate alignment of transcriptomes in the presence of insertions, deletions and gene fusions. Genome Biol..

[90] Love M.I., Huber W., Anders S. (2014). Moderated estimation of fold change and dispersion for RNA-seq data with DESeq2. Genome Biol..

[91] Ross P.L., Huang Y.N., Marchese J.N., Williamson B., Parker K., Hattan S., Khainovski N., Pillai S., Dey S., Daniels S. (2004). Multiplexed protein quantitation in *Saccharomyces cerevisiae* using amine-reactive isobaric tagging reagents. Mol. Cell. Proteom..

[92] Wiśniewski J.R., Zougman A., Nagaraj N., Mann M. (2009). Universal sample preparation method for proteome analysis. Nat. Methods.

[93] Stefan G., Miguel G.G.J., Javier T., Williams T.D., Nagaraj S.H., José N.M., Montserrat R., Manuel T., Joaquín D., Ana C. (2008). High-throughput functional annotation and data mining with the Blast2GO suite. Nucleic Acids Res..

[94] Kanehisa M., Goto S., Sato Y., Furumichi M., Tanabe M. (2011). KEGG for integration and interpretation of large-scale molecular data sets. Nucleic Acids Res..

[95] Decourcelle M., Perez-Fons L., Baulande S., Steiger S., Couvelard L., Hem S., Zhu C., Capell T., Christou P., Fraser P. (2015). Combined transcript, proteome, and metabolite analysis of transgenic maize seeds engineered for enhanced carotenoid synthesis reveals pleotropic effects in core metabolism. J. Exp. Bot..

[96] Smart K.F., Aggio R.B.M., Van Houtte J.R., Villas-Bôas S.G. (2010). Analytical platform for metabolome analysis of microbial cells using methyl chloroformate derivatization followed by gas chromatography–mass spectrometry. Nat. Protoc..

[97] Bölling C., Fiehn O. (2005). Metabolite profiling of Chlamydomonas reinhardtii under nutrient deprivation. Plant Physiol..

[98] Core R., Null R.D.C.T., Team R., Team T.S.U.D.I.S., Coreteam R. (2011). R: A Language and Environment for Statistical Computating. Computing.

[99] Zeng Y., Tan X., Lin Z., Long H., Wang B., Li Z., Zhen Y. (2015). A fructose-1,6-biphosphate aldolase gene from *Camellia oleifera*: Molecular characterization and impact on salt stress tolerance. Mol. Breed..

[100] Livak K., Schmittgen T. (2000). Analysis of Relative Gene Expression Data Using Real-Time Quantitative PCR and the 2^-^^△△Ct^ Method. Methods.

