# Supplementary material for: In-Depth Understanding of Camellia oleifera Self-Incompatibility by Comparative Transcriptome, Proteome and Metabolome
Source: Int J Mol Sci. 2020 Feb 26;21(5):1600. doi: 10.3390/ijms21051600 (PMC7084461; doi:10.3390/ijms21051600)
Supplement: Supplementary file 1 [file ijms-21-01600-s001.zip › Supplementary materials/Figure S1B.pdf]

B

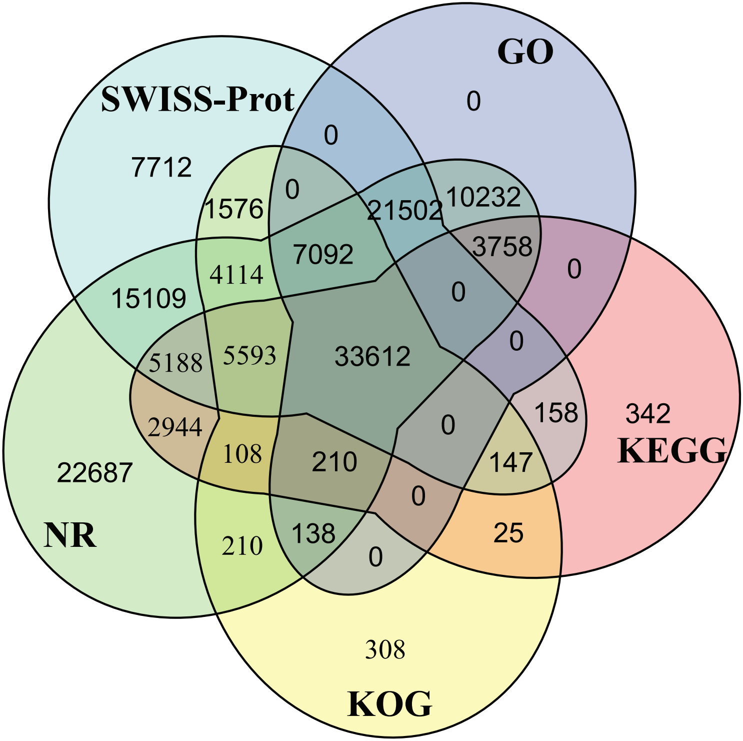

GO
SWISS-Prot
0
0
7712
0
10232
1576
21502
3758
7092
0
4114
15109
0
0
5188
5593
33612
158
342
2944
0
KEGG
108
210
147
22687
0
NR
138
25
210
0
308
KOG
