# Supplementary material for: In-Depth Understanding of Camellia oleifera Self-Incompatibility by Comparative Transcriptome, Proteome and Metabolome
Source: Int J Mol Sci. 2020 Feb 26;21(5):1600. doi: 10.3390/ijms21051600 (PMC7084461; doi:10.3390/ijms21051600)
Supplement: Supplementary file 1 [file ijms-21-01600-s001.zip › Supplementary materials/Figure S1C.pdf]

C

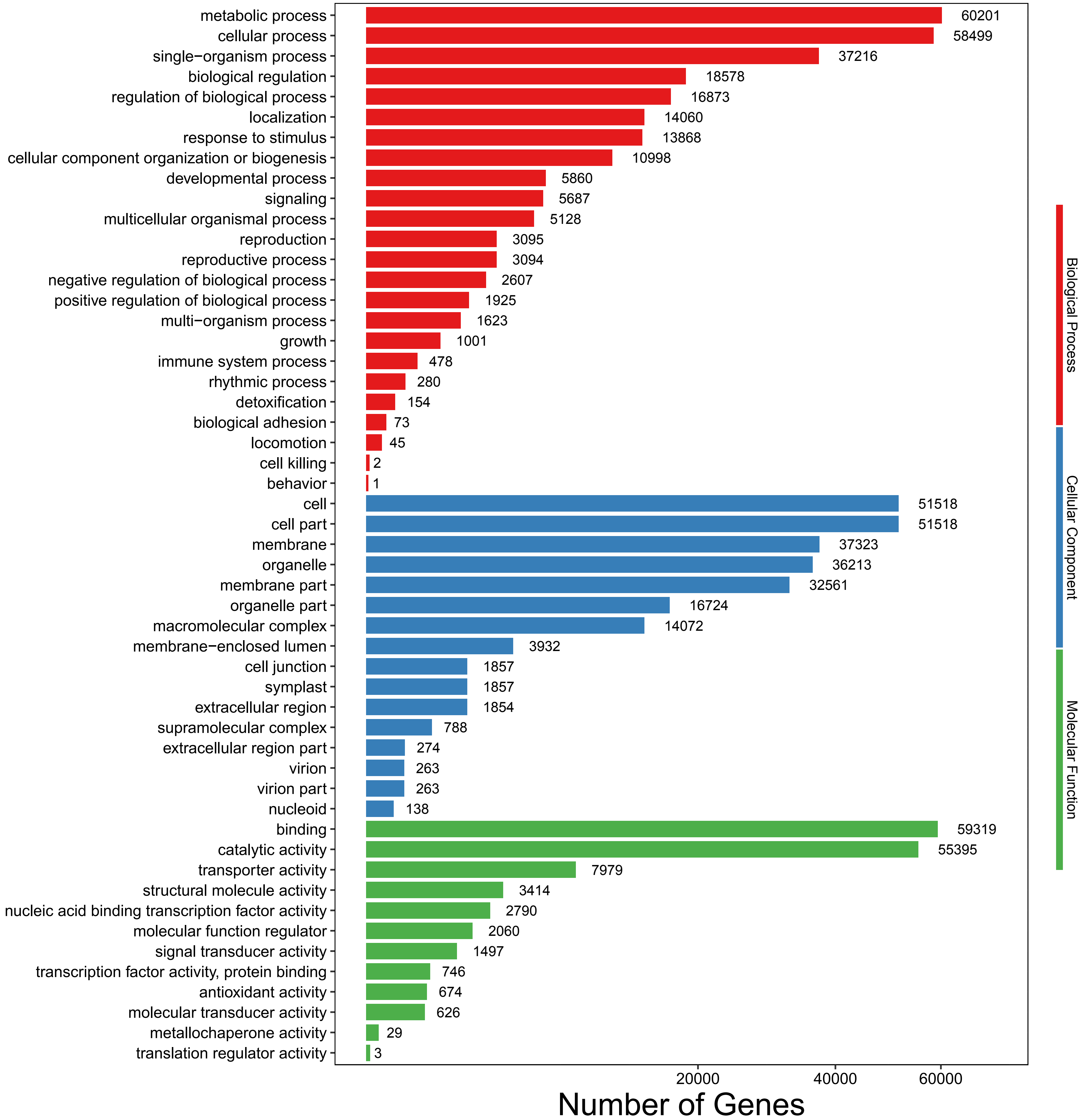
metabolic process 60201
cellular process 58499
single−organism process 37216
biological regulation 18578
regulation of biological process 16873
localization 14060
response to stimulus 13868
cellular component organization or biogenesis 10998
developmental process 5860
signaling 5687
multicellular organismal process 5128
reproduction 3095
reproductive process 3094
negative regulation of biological process 2607
positive regulation of biological process 1925
multi−organism process 1623
growth 1001
immune system process 478
rhythmic process 280
detoxification 154
biological adhesion 73
locomotion 45
cell killing 2
behavior 1
cell 51518
cell part 51518
membrane 37323
organelle 36213
membrane part 32561
organelle part 16724
macromolecular complex 14072
membrane−enclosed lumen 3932
cell junction 1857
symplast 1857
extracellular region 1854
supramolecular complex 788
extracellular region part 274
virion 263
virion part 263
nucleoid 138
binding 59319
catalytic activity 55395
transporter activity 7979
structural molecule activity 3414
nucleic acid binding transcription factor activity 2790
molecular function regulator 2060
signal transducer activity 1497
transcription factor activity, protein binding 746
antioxidant activity 674
molecular transducer activity 626
metallochaperone activity 29
translation regulator activity 3
20000
40000
60000
Number of Genes
Biological Process
Cellular Component
Molecular Function
