# Supplementary material for: In-Depth Understanding of Camellia oleifera Self-Incompatibility by Comparative Transcriptome, Proteome and Metabolome
Source: Int J Mol Sci. 2020 Feb 26;21(5):1600. doi: 10.3390/ijms21051600 (PMC7084461; doi:10.3390/ijms21051600)
Supplement: Supplementary file 1 [file ijms-21-01600-s001.zip › Supplementary materials/Figure S1D.pdf]

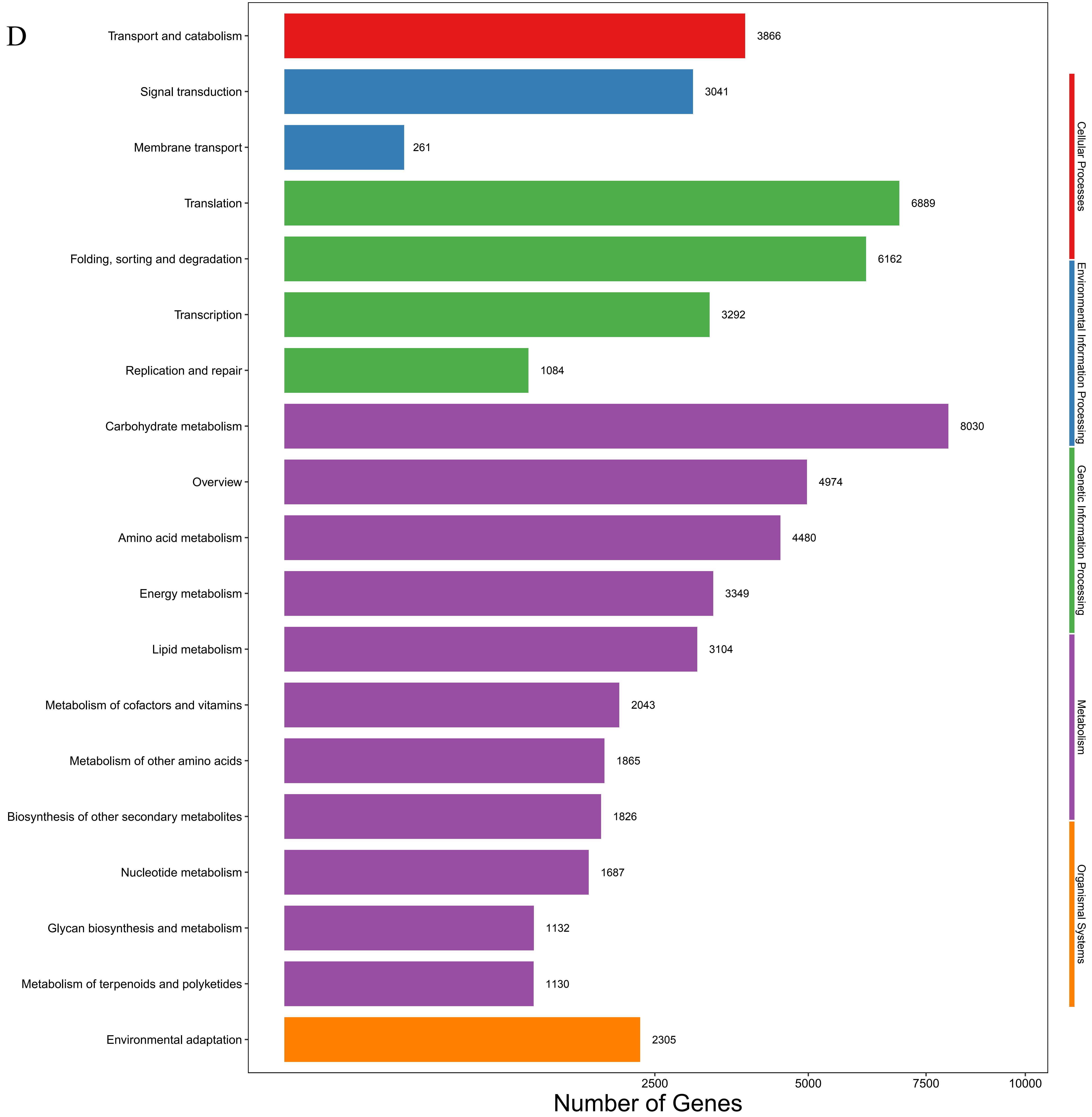

D
Transport and catabolism
3866
Signal transduction
3041
Membrane transport
261
Translation
6889
Folding, sorting and degradation
6162
Transcription
3292
Replication and repair
1084
Carbohydrate metabolism
8030
Overview
4974
Amino acid metabolism
4480
Energy metabolism
3349
Lipid metabolism
3104
Metabolism of cofactors and vitamins
2043
Metabolism of other amino acids
1865
Biosynthesis of other secondary metabolites
1826
Nucleotide metabolism
1687
Glycan biosynthesis and metabolism
1132
Metabolism of terpenoids and polyketides
1130
Environmental adaptation
2305
2500
5000
7500
10000
Number of Genes
Cellular Processes
Environmental Information Processing
Genetic Information Processing
Metabolism
Organismal Systems
