# Supplementary material for: In-Depth Understanding of Camellia oleifera Self-Incompatibility by Comparative Transcriptome, Proteome and Metabolome
Source: Int J Mol Sci. 2020 Feb 26;21(5):1600. doi: 10.3390/ijms21051600 (PMC7084461; doi:10.3390/ijms21051600)
Supplement: Supplementary file 1 [file ijms-21-01600-s001.zip › Supplementary materials/Figure S1F.pdf]

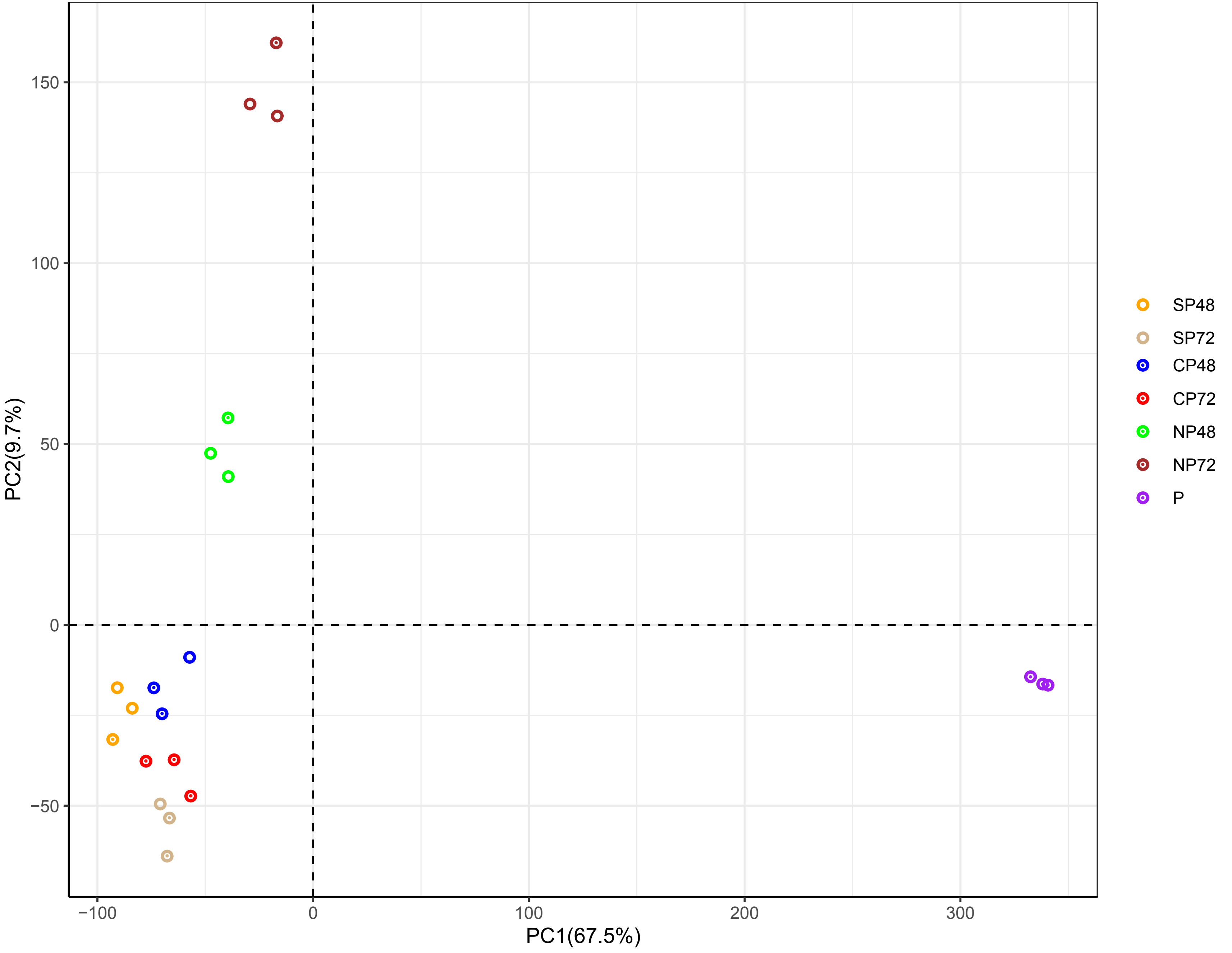

150
100
50
0
−50
−100
0
100
200
300
PC1(67.5%)
PC2(9.7%)
SP48
SP72
CP48
CP72
NP48
NP72
P
