# Supplementary material for: In-Depth Understanding of Camellia oleifera Self-Incompatibility by Comparative Transcriptome, Proteome and Metabolome
Source: Int J Mol Sci. 2020 Feb 26;21(5):1600. doi: 10.3390/ijms21051600 (PMC7084461; doi:10.3390/ijms21051600)
Supplement: Supplementary file 1 [file ijms-21-01600-s001.zip › Supplementary materials/Figure S2.pdf]

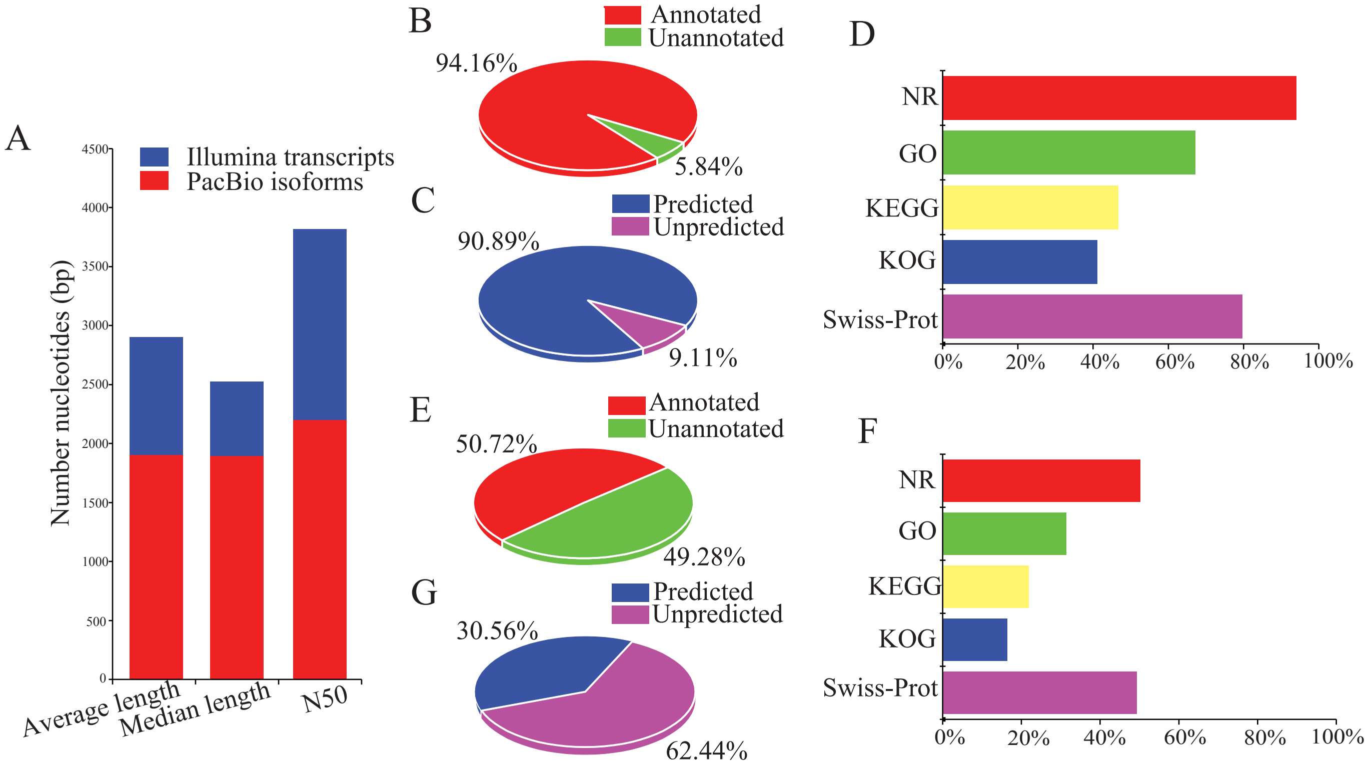
A
Number nucleotides (bp)
4500
4000
3500
3000
2500
2000
1500
1000
500
0
Illumina transcripts
PacBio isoforms
Average length
Median length
N50
B
Annotated
Unannotated
94.16%
5.84%
C
Predicted
Unpredicted
90.89%
9.11%
E
Annotated
Unannotated
50.72%
49.28%
G
Predicted
Unpredicted
30.56%
62.44%
D
NR
GO
KEGG
KOG
Swiss-Prot
0%
20%
40%
60%
80%
100%
F
NR
GO
KEGG
KOG
Swiss-Prot
0%
20%
40%
60%
80%
100%
