# Supplementary material for: In-Depth Understanding of Camellia oleifera Self-Incompatibility by Comparative Transcriptome, Proteome and Metabolome
Source: Int J Mol Sci. 2020 Feb 26;21(5):1600. doi: 10.3390/ijms21051600 (PMC7084461; doi:10.3390/ijms21051600)
Supplement: Supplementary file 1 [file ijms-21-01600-s001.zip › Supplementary materials/Figure S4.pdf]

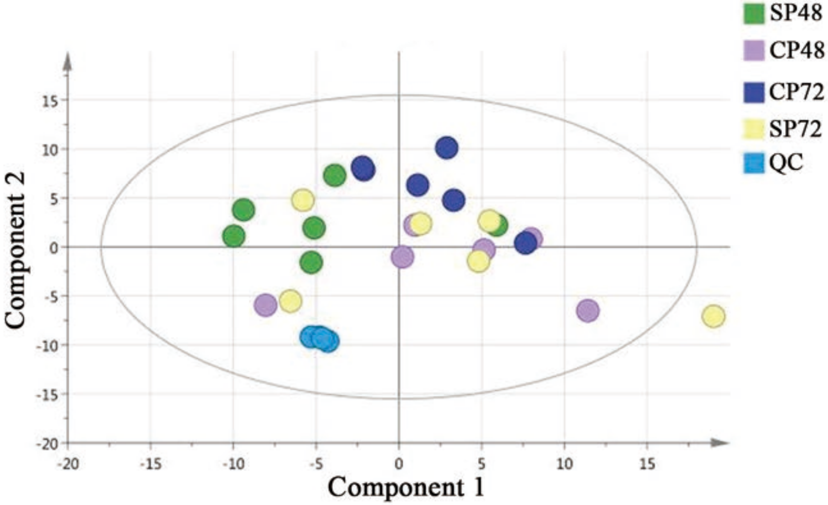
SP48
CP48
CP72
SP72
QC
Component 2
Component 1
15
10
5
0
-5
-10
-15
-20
-20
-15
-10
-5
0
5
10
15
