# Supplementary material for: In-Depth Understanding of Camellia oleifera Self-Incompatibility by Comparative Transcriptome, Proteome and Metabolome
Source: Int J Mol Sci. 2020 Feb 26;21(5):1600. doi: 10.3390/ijms21051600 (PMC7084461; doi:10.3390/ijms21051600)
Supplement: Supplementary file 1 [file ijms-21-01600-s001.zip › Supplementary materials/Figure S5.pdf]

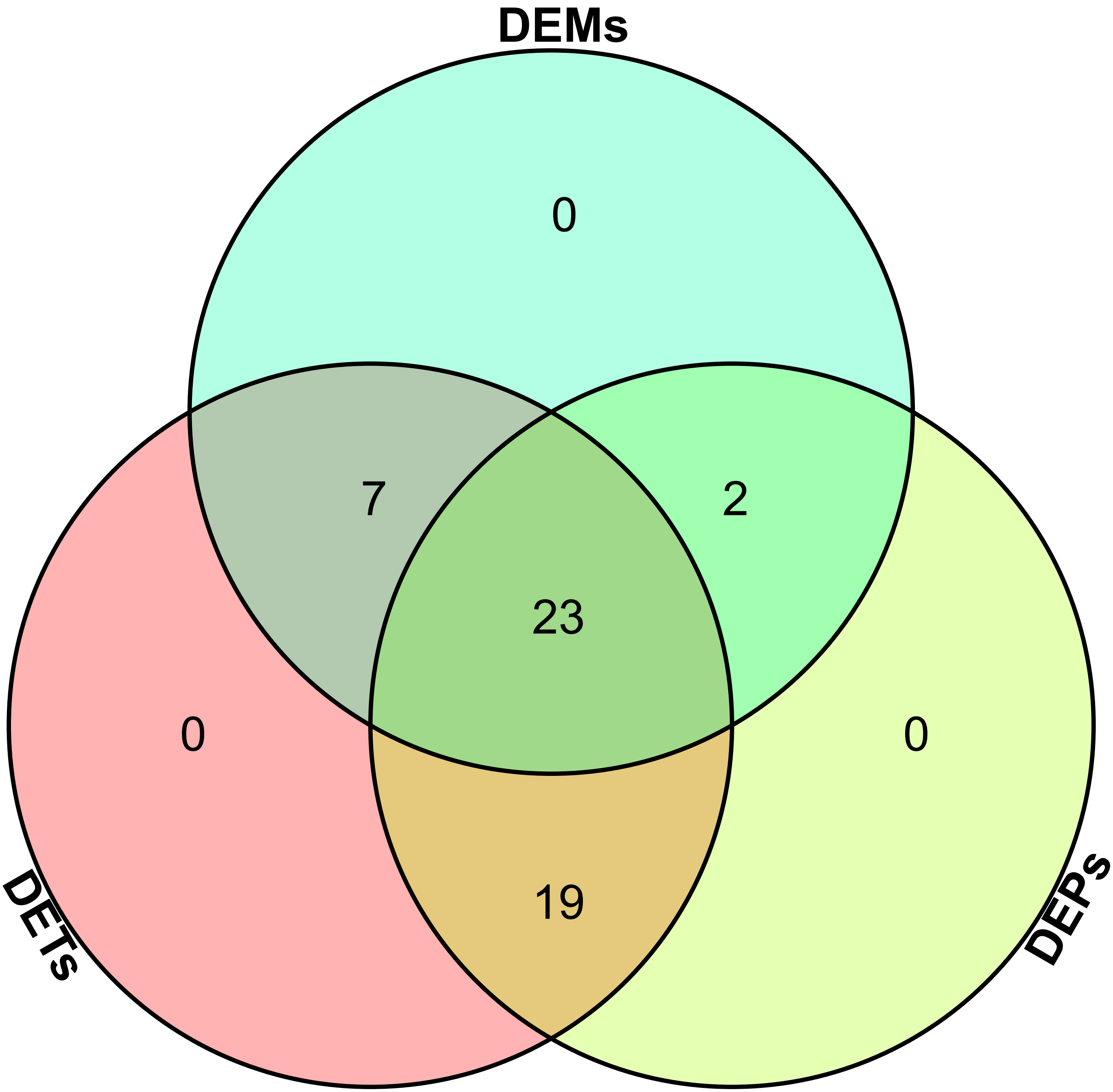
DEMs
0
7
2
23
0
0
19
DETs
DEPs
